# The long noncoding RNA THBS1-AS1 promotes cardiac fibroblast activation in cardiac fibrosis by regulating TGFBR1

**DOI:** 10.1172/jci.insight.160745

**Published:** 2023-03-22

**Authors:** Junteng Zhou, Geer Tian, Yue Quan, Qihang Kong, Fangyang Huang, Junli Li, Wenchao Wu, Yong Tang, Zhichao Zhou, Xiaojing Liu

**Affiliations:** 1 Laboratory of Cardiovascular Diseases, Regenerative Medicine Research Center,; 2 Health Management Center, General Practice Medical Center, and; 3 Department of Cardiology, West China Hospital, Sichuan University, Chengdu, China.; 4 International Joint Research Centre on Purinergic Signaling, Chengdu University of Traditional Chinese Medicine, Chengdu, China.; 5 Acupuncture & Chronobiology Key Laboratory of Sichuan Province, Chengdu, China.; 6 Division of Cardiology, Department of Medicine Solna, Karolinska University Hospital, Karolinska Institutet, Stockholm, Sweden.

**Keywords:** Cardiology, Cardiovascular disease, Fibrosis, Noncoding RNAs

## Abstract

Cardiac fibrosis is associated with an adverse prognosis in cardiovascular disease that results in a decreased cardiac compliance and, ultimately, heart failure. Recent studies have identified the role of long noncoding RNA (lncRNA) in cardiac fibrosis. However, the functions of many lncRNAs in cardiac fibrosis remain to be characterized. Through a whole-transcriptome sequencing and bioinformatics analysis on a mouse model of pressure overload–induced cardiac fibrosis, we screened a key lncRNA termed thrombospondin 1 antisense 1 (THBS1-AS1), which was positively associated with cardiac fibrosis. In vitro functional studies demonstrated that the silencing of THBS1-AS1 ameliorated TGF-β1 effects on cardiac fibroblast (CF) activation, and the overexpression of THBS1-AS1 displayed the opposite effect. A mechanistic study revealed that THBS1-AS1 could sponge miR-221/222 to regulate the expression of TGFBR1. Moreover, under TGF-β1 stimulation, the forced expression of miR-221/222 or the knockdown TGFBR1 significantly reversed the THBS1-AS1 overexpression induced by further CF activation. In vivo, specific knockdown of THBS1-AS1 in activated CFs significantly alleviated transverse aorta constriction–induced (TAC-induced) cardiac fibrosis in mice. Finally, we demonstrated that the human THBS1-AS1 can also affect the activation of CFs by regulating TGFBR1. In conclusion, this study reveals that lncRNA THBS1-AS1 is a potentially novel regulator of cardiac fibrosis and may serve as a target for the treatment of cardiac fibrosis.

## Introduction

Cardiac fibrosis, associated with an adverse prognosis in cardiovascular disease, is one of the most common pathophysiological processes induced by acute or chronic stimuli such as myocardial infarction and hypertension (1, 2). Following the mechanical stimulation, pressure/volume overload, humoral, and other pathological factors, cardiac fibroblasts (CFs) proliferate and transdifferentiate to myofibroblasts, resulting in the excessive secretion of the extracellular matrix (ECM), decreased cardiac compliance and cardiac remodeling, and ultimately heart failure (3, 4). Currently, effective clinical treatments for CF activation and fibrosis are lacking. Therefore, discovering key molecules for cardiac fibrosis and elucidating the mechanism are of great value for cardiovascular treatment.

Emerging evidence has explored long noncoding RNA (lncRNA) as a regulator and a potential therapeutic target for various cardiovascular diseases (5–7). LncRNA is a noncoding RNA class with a length of more than 200 nucleotides, which can affect the chromatin structure and the functions of transcription factors through protein binding (8). LncRNA also binds to microRNA (miRNA) or mRNA by its linear structure, affecting mRNA translation, splicing, degradation, and other processes (9). Due to some inherent difficulties, such as its conservatism, secondary structure effect, and cell type–specific expression profile, only few lncRNAs with definite biological functions related to cardiac fibrosis have been identified. However, the key lncRNAs that are involved in cardiac fibrosis remain to be determined.

The TGF-β signaling pathway plays an important role in cardiac fibrosis by regulating cell proliferation differentiation, and apoptosis (10). However, a broad inhibitor of the TGF-β signaling pathway such as activin receptor-like kinase 5 (ALK5) may not be very effective and will produce severe cardiotoxicity, due to its function involved in physiology (11, 12). The modulation of the TGF-β signaling pathway by the endogenous lncRNA may be a novel therapeutic strategy without inducing potential side effects. However, very few studies have explored the regulatory relationship between lncRNA and TGF-β signaling in cardiac fibrosis.

Through a whole-transcriptome sequencing and bioinformatics analysis, we identified a key lncRNA named thrombospondin 1 antisense 1 (THBS1-AS1, ENST00000616754.1), which was positively associated with TGF-β signaling in the TAC-induced cardiac fibrosis model. We found that lncRNA THBS1-AS1 enhanced the progression of CF activation via regulating the TGFBR1 in both mouse and human CFs (HCFs). Also, the knockdown of THBS1-AS1 could improve cardiac fibrosis and cardiac function in vivo. Our findings suggest that lncRNA THBS1-AS1 is a potential target for the treatment of cardiac fibrosis.

## Results

### Evaluation of mouse cardiac fibrosis model and RNA-Seq in the left ventricle.

To observe the dynamic changes of cardiac anatomy and function in different periods in cardiac fibrosis, we constructed a pressure overload–induced cardiac fibrosis mouse model, a well-established model for the study of cardiac fibrosis and hypertrophy (13, 14), at 2, 4, and 8 weeks following transverse aorta constriction (TAC). When echocardiography was performed, there was no significant difference in heart rates between Sham, TAC_2w, TAC_4w, and TAC_8w group mice (Table 1); TAC_2w, TAC_4w, and TAC_8 represent 2 weeks, 4 weeks, and 8 weeks after TAC, respectively. The cardiac function and structure deteriorated over time following TAC, as evidenced by a significantly lower left ventricular ejection fraction (EF) and significantly higher left ventricular internal diameter in diastole (LVIDd), left ventricular internal diameter in systole (LVIDs), interventricular septum in diastole (IVSd), LV end diastolic volumes (LV Vol;d), and left ventricular posterior wall diameter (LVPWd) (Table 1 and Supplemental Figure 1, A and B; supplemental material available online with this article; https://doi.org/10.1172/jci.insight.160745DS1). In addition, there was an increased marked collagen deposition after 2, 4, and 8 weeks following TAC (Supplemental Figure 1, C and E). Likewise, wheat germ agglutinin (WGA) indicated an increased cross-sectional area of cardiomyocytes following TAC compared with Sham mice (Supplemental Figure 1, D and E). These changes were also accompanied by liver function injury and a slight deterioration of renal function (Supplemental Table 5).

**Figure 1:**
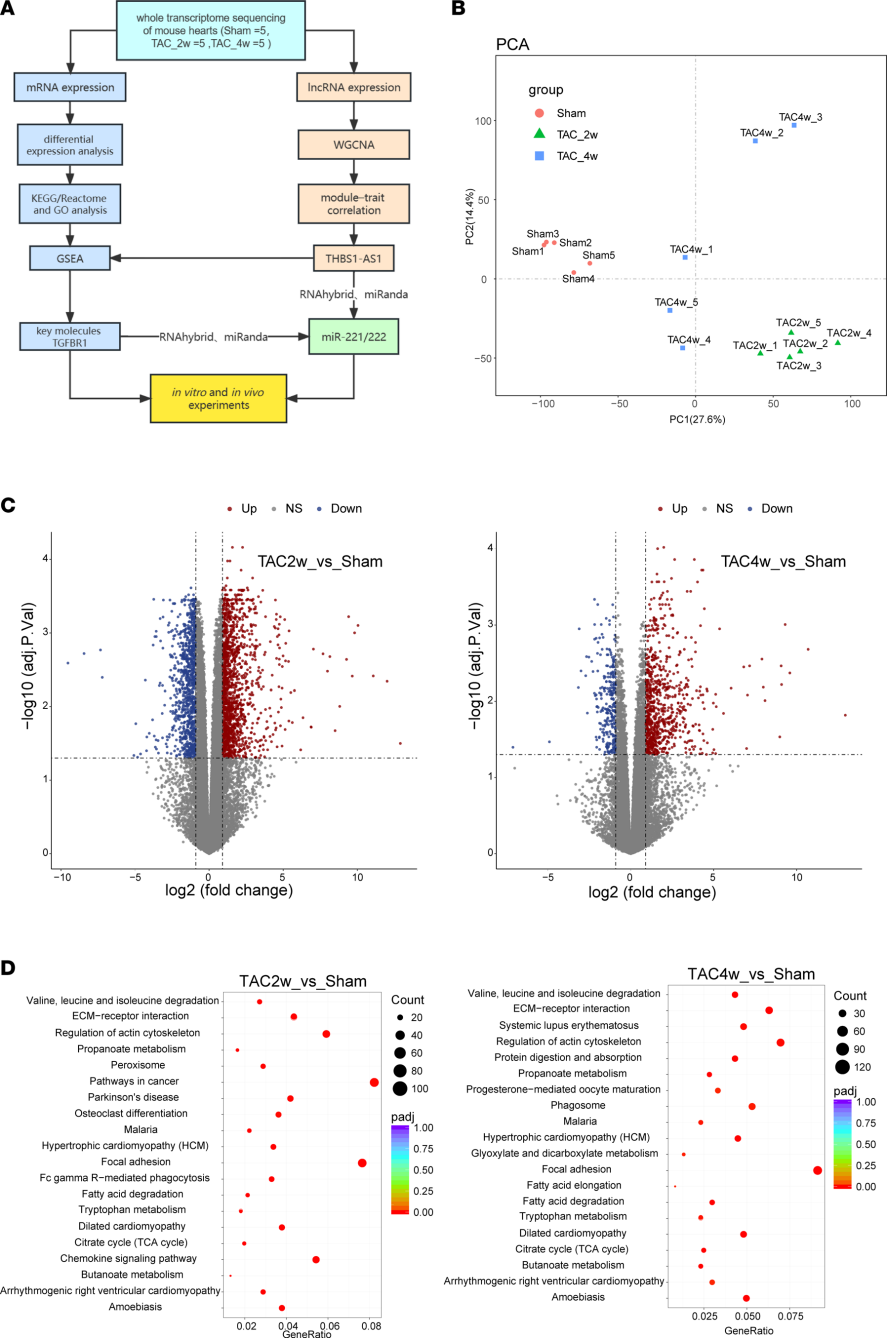
Evaluation of mouse cardiac fibrosis model and RNA-Seq. (**A**) The workflow of experimental strategy in the mouse model of TAC. (**B**) PCA plots and hierarchical 2D clustering of gene expression in the Sham, TAC_2w, and TAC_4w mice. (**C**) The volcano map showing differentially expressed mRNAs in the heart 2 and 4 weeks after TAC and Sham-operated mice (*n* = 5 in each group). (**D**) The KEGG pathway enrichment analysis of differential genes. The graph of *P* values from the pathway enrichment analysis. Results are presented as means ± SEM.

Previous studies have shown that a mouse will develop adaptive cardiac hypertrophy at 2 weeks and progress to heart failure/cardiac fibrosis at 4 weeks in response to pressure overload (15). Therefore, we selected mice from both Sham and TAC groups at 2 and 4 weeks — representing the normal group, adaptive hypertrophy, and heart failure/cardiac fibrosis stages — for the transcriptome sequencing (Figure 1A). A cluster analysis shows tight clustering of each experimental condition (Figure 1B). Compared with the Sham group, there were 1,567 upregulated and 970 downregulated genes detected in the TAC_2w group and 804 upregulated and 298 downregulated genes in the TAC_4w group (Figure 1C). Differential genes in TAC_2w versus Sham and TAC_4w versus Sham were enriched in several pathways related to fibrosis formation such as the ECM, the ECM organization, the ECM-receptor interaction, focal adhesion, collagen formation, the assembly of collagen fibrils, and other multimeric structures (Figure 1D and Supplemental Tables 6 and 7).

### Identification of THBS1-AS1 as a potentially novel lncRNA in cardiac fibrosis.

**Figure 2:**
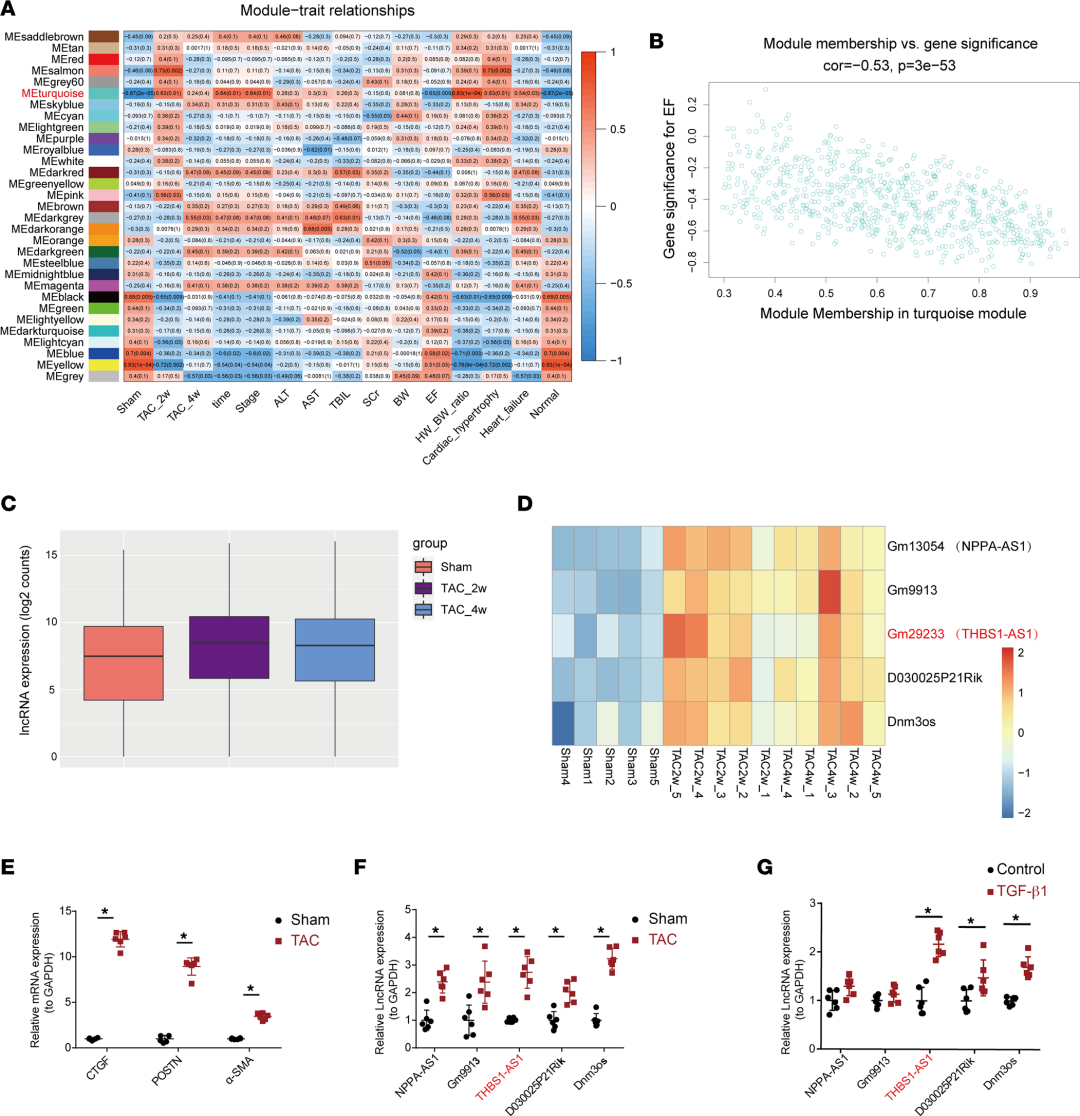
The upregulated expression of lncRNA Gm29233 (THBS1-AS1) in the hearts of mice subjected to TAC and in activated CFs. (**A**) The module-trait associations. Each row refers to an eigengene, and each column corresponds to a clinical trait. (**B**) The scatter plot of turquoise module eigengenes. (**C** and **D**) The expression pattern of all of the lncRNA, Gm13054 (NPPA-AS1), Gm9913, Gm29233 (THBS1-AS1), D030025P21Rik, and Dnm3os among the Sham, TAC_2w, and TAC_4w groups. (**E**) The gene expression of CTGF, Periostin, and α-SMA in mouse hearts by qPCR (*n* = 6). (**F**) The gene expression of Gm13054 (NPPA-AS1), Gm9913, Gm29233 (THBS1-AS1), D030025P21Rik, and Dnm3os in mouse hearts by qPCR (*n* = 6). (**G**) The gene expression of lncRNA, Gm13054, Gm9913, Gm29233, D030025P21Rik, and Dnm3os stimulated with TGF-β1 for 24 hours (*n* = 6). An unpaired, 2-tailed *t* test was used. The results are presented as means ± SEM; *P < 0.05 versus the control.

To find the molecules that play key roles in a large number of genes, we used a weighted gene coexpression network analysis (WGCNA) to screen for phenotype-associated lncRNAs. After quality control of the samples, lncRNA was used to construct the coexpression network by WGCNA. We chose the smallest soft threshold power to 9 (scale-free *R*^2^ > 0.85) and set the height to 0.25 for the next analysis (Supplemental Figure 2A). Then, we constructed a gene clustering tree based on the gene-to-gene similarity and divided the modules according to the clustering relationship between genes (Supplemental Figure 2B). Genes that could not be aggregated to any of these modules were assigned to the gray module, which was not used for the subsequent analysis (Supplemental Figure 2B). A heatmap visualized the correlations between modules and clinical traits (Figure 2A). Interestingly, the results showed that the turquoise module was significantly positively correlated with the stage of the sample, heart weight/body weight ratio (HW/BW) ratio, cardiac hypertrophy, and heart failure (Pearson coefficients = 0.64, 0.83, 0.63, and 0.54, respectively; *P* < 0.05) and negatively correlated with EF (Pearson coefficient = –0.65, *P* < 0.05) (Figure 2A). As shown in Figure 2B, the turquoise module membership (MM) and gene significance for EF were significantly correlated. By screening the MM values and gene significance (GS) in the turquoise module, we selected Gm13054, Gm9913, Gm29233, D030025P21Rik, and Dnm3os for further analysis (Figure 2, C and D). The overall expression pattern of the turquoise module genes is shown in Figure 2C. Specifically, as shown in Figure 2, C and D, it was at a low level in the Sham group; the overall expression levels were increased in the TAC_2w and TAC_4w group.

In order to confirm the above results, we validated the expressions of lncRNAs in vivo and in vitro. Compared with the Sham group, the expression of lncRNA Gm13054 (NPPA-AS1), Gm9913, Gm29233 (THBS1-AS1), D030025P21Rik, and Dnm3os was significantly increased in the heart tissues of the TAC_4w group, accompanied by the elevation of mRNA expression levels for fibrosis markers, α-smooth muscle actin (α-SMA), POSTN, and connective tissue growth factor (CTGF), respectively (Figure 2, E and F). Then, we used TGF-β1 (10 ng/mL) to induce fibroblast differentiation to contractile myofibroblasts for 24 hours (Supplemental Figure 3, A–D). The expressions of THBS1-AS1, D030025P21Rik, and Dnm3os were increased in the activated CFs induced by TGF-β1 compared with the control group (Figure 2G), and there were trends of increasing expressions of Gm29233 and Dnm3os after 3, 6, 12, and 24 hours of stimulation (Supplemental Figure 4). Given that Dnm3os has been extensively studied in diseases such as pulmonary fibrosis (16) and heart failure (17), we conducted an in-depth study for Gm29233, due to its unreported role.

The lncRNA Gm29233 (ENSMUSG0000010185) is located at the negative strand of chr2:118123451-118124047 with a genomic length of 597 bp, and its corresponding transcript is ENSMUST00000190311 with a length of 281 bp, located in the antisense strand of THBS1. Thus, we named the uncharacterized lncRNA THBS1-AS1. Its position in the genome is shown in Supplemental Figure 5. To further define the molecular mechanism driving the THBS1-AS1 expression in cardiac fibrosis, we comprehensively analyzed several databases, including the mouse transcription factor database (18), our RNA-Seq data generated from primary mouse CFs exposed to TGF-β1/control (24 hours) (PRJNA838231), or mouse left ventricles after 2 or 4 weeks of Sham/TAC treatment (PRJNA787574). Among the shared upregulated DEGs in activated CFs and cardiac fibrosis, there were 4 top-changed transcription factors, namely Meox1, Runx1, Arntl, and Egr2 (Supplemental Figure 6, A–C). We then chose to focus on Meox1, whose fold-change was the most evident. Furthermore, using the JASPAR programs (http://jaspar.genereg.net), we analyzed the 2,000 upstream and 100 downstream of the THBS1-AS1 transcription start site to identify whether Meox1 had potential binding sites. Interestingly, 16 bindings site were predicted by JASPAR (Supplemental Table 8). In addition, ChIP-Seq data of Meox1 and H3K27ac in activated CFs (GSE155882) showed an enrichment in the region proximal to the THBS1-AS1 transcriptional start site (Supplemental Figure 6, D and E). Indeed, Meox1 was significantly upregulated in activated CFs and TAC mice compared with the control (Supplemental Figure 6, F and G). Furthermore, we found that THBS1-AS1 were obviously downregulated after the Meox1 knockdown in the CF treated with TGF-β1 (Supplemental Figure 6H). Collectively, these data illustrate that Meox1 regulated THBS1-AS1 expression at the transcriptional level in the development of cardiac fibrosis.

### Functional role of THBS1-AS1 in CF activation.

**Figure 3:**
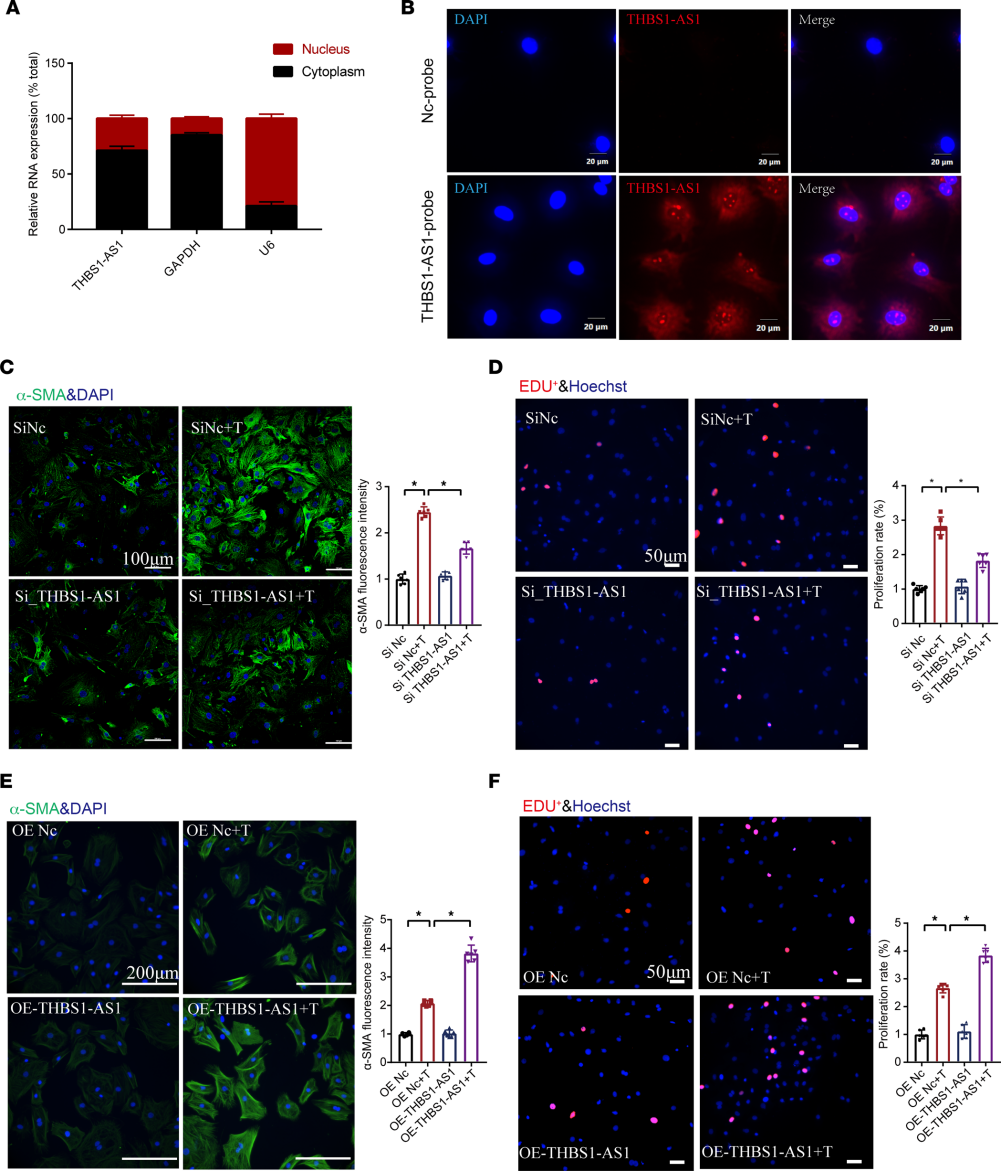
The role of THBS1-AS1 on cardiac fibroblast activation. (**A**) LncRNA THBS1-AS1 is abundant in the cytoplasm of cardiac fibroblasts. GAPDH mRNA and U6 were applied as positive controls in the cytoplasm and nucleus, respectively. (**B**) RNA FISH for THBS1-AS1 in cardiac fibroblasts. THBS1-AS1 was shown in red fluorescence (Cy3-labeled), and the nuclei were stained with DAPI. (**C**) Immunoﬂuorescence images showing α-SMA expression in cardiac fibroblasts. The images show staining for α-SMA (green) and nuclei DAPI (blue) (*n* = 6). (**D**) The EdU staining used to detect cell proliferation. The cell nuclei are stained blue (DAPI), and the EdU^+^ nuclei are stained red (*n* = 6). (**E**) Immunoﬂuorescence images showing α-SMA expression in cardiac fibroblasts (*n* = 6). (**F**) The EdU staining used to detect cell proliferation (*n* = 6). One-way ANOVA, followed by a Bonferroni post hoc test, was used. The results are presented as means ± SEM; *P < 0.05.

For the investigation of the subcellular locations, we extracted nuclear and cytoplasmic RNA from the primary culture of mouse neonatal CFs and examined the nucleoplasmic expression of THBS1-AS1. As shown in Figure 3A, THBS1-AS1 was about 72% distributed in the cytoplasm, while GAPDH was mainly expressed in the cytoplasm and U6 mainly in the nucleus. Of interest, using RNA FISH, we observed that Cy3 (red fluorescence) was mainly located in the cytoplasm (Figure 3B), suggesting that lncRNA THBS1-AS1 may mainly affect the expression level of genes by regulating the stability of mRNAs.

To determine the functional role of lncRNA THBS1-AS1 in the activation of CFs, we performed a gain- and loss-of-function assay. We used 3 different siRNAs to specifically silence the expression of THBS1-AS1 in CFs. The results showed that the second of three siRNA (siRNA 2), which we used for the following study, effectively suppressed approximately 68% of the expression of lncRNA THBS1-AS1 compared with the negative control (Nc) (Supplemental Figure 7A). As shown in Supplemental Figure 7B, compared with the TGF-β1–treated Nc group (siNc+T group), silencing lncRNA THBS1-AS1 followed by TGF-β1 stimulation for 24 hours (si-THBS1-AS1+T) decreased the CF activation markers POSTN, CTGF, and α-SMA. Immunofluorescence results showed that the knockdown of lncRNA THBS1-AS1 decreased the fluorescence intensity of α-SMA (Figure 3C). Scratch (Supplemental Figure 7C), collagen contraction (Supplemental Figure 7D), and EdU assay (Figure 3D) also showed that the knockdown of lncRNA THBS1-AS1 significantly inhibited the migration, collagen contraction, and proliferation ability of TGF-β1–activated cardiac myofibroblasts. In addition, THBS1-AS1 knockdown by antisense oligonucleotides (ASO) was also used to investigate the role of THBS1-AS1 in activated CFs. As expected, the same antifibrosis effects were observed by an ASO-mediated knockdown of THBS1-AS1 in activated CFs (Supplemental Figure 8, A and B).

For the overexpression (OE) of THBS1-AS1, adenovirus at 200 MOI was used to transfect CFs. Quantitative PCR (qPCR) results showed a 134-fold increase in the expression level of lncRNA THBS1-AS1 (Supplemental Figure 9A). Of note, the adenovirus-specific OE of lncRNA THBS1-AS1 promoted TGF-β1–induced activation of CFs and enhanced their proliferation, migration, and collagen contraction (Figure 3, E and F, and Supplemental Figure 9, B–D).

**Figure 4:**
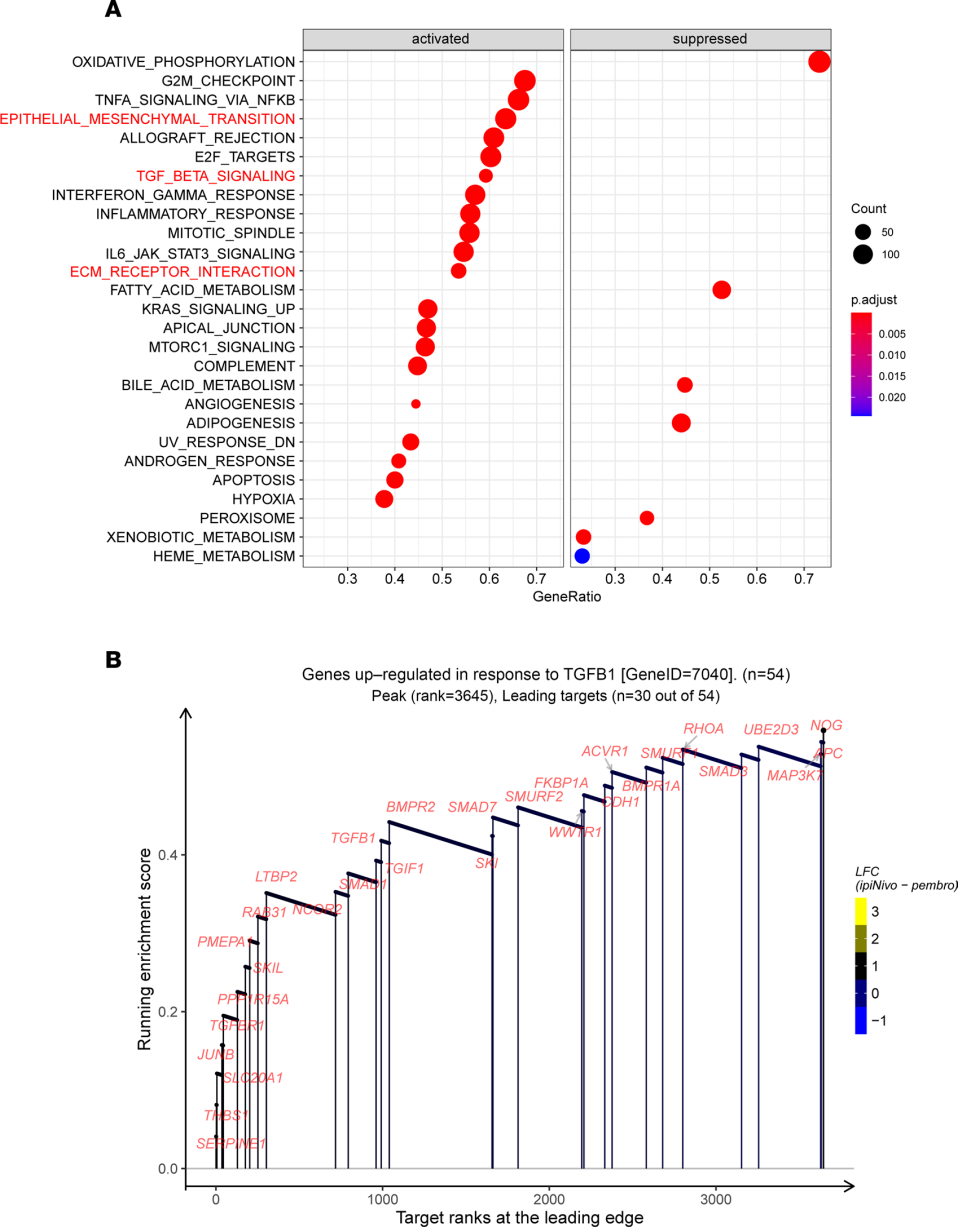
The potential regulation mechanisms of THBS1-AS1 in the fibrosis process. (**A**) The rank-based gene set enrichment analysis of genes significantly associated with THBS1-AS1 identifies the top 20 enriched pathways preferentially induced or suppressed. (**B**) The enrichment plots showing rank-based genes related to the TGF-β signaling pathway in pressure overload–induced cardiac fibrosis.

To further explore the function of this lncRNA, we conducted a gene set enrichment analysis (GSEA) to explore the potential downstream pathways of THBS1-AS1. Figure 4A demonstrates the top 20 pathways enriched by GSEA, where oxidative phosphorylation, fatty acid metabolism, bile acid metabolism, lipogenesis, peroxisome, and xenobiotic metabolic pathways were significantly inhibited. In addition, the NF-κB–mediated tumor necrosis factor signaling pathway, the epithelial-mesenchymal transition, the TGF-β, ECM junctions, the cell cycle, and the inflammatory response signaling pathways were significantly activated. All of these changes are closely related to the cardiac fibrosis process. Among them, the TGF-β signaling pathway, the epithelial-mesenchymal transition, wound healing, the fibrosis and metastasis signaling pathway, and the ECM junctional signaling pathway are most noteworthy to us (Figure 4B and Supplemental Figure 10, A and B). We showed the genes with the largest enrichment contribution and found that THBS1-AS1 was significantly associated with some fibrosis markers, such as TGFBR1, POSTN, FN1, Col4a1, and LTBP2.

### The regulation of CF activation by lncRNA THBS1-AS1 via the miR-221/222-TGFBR1 axis.

**Figure 5:**
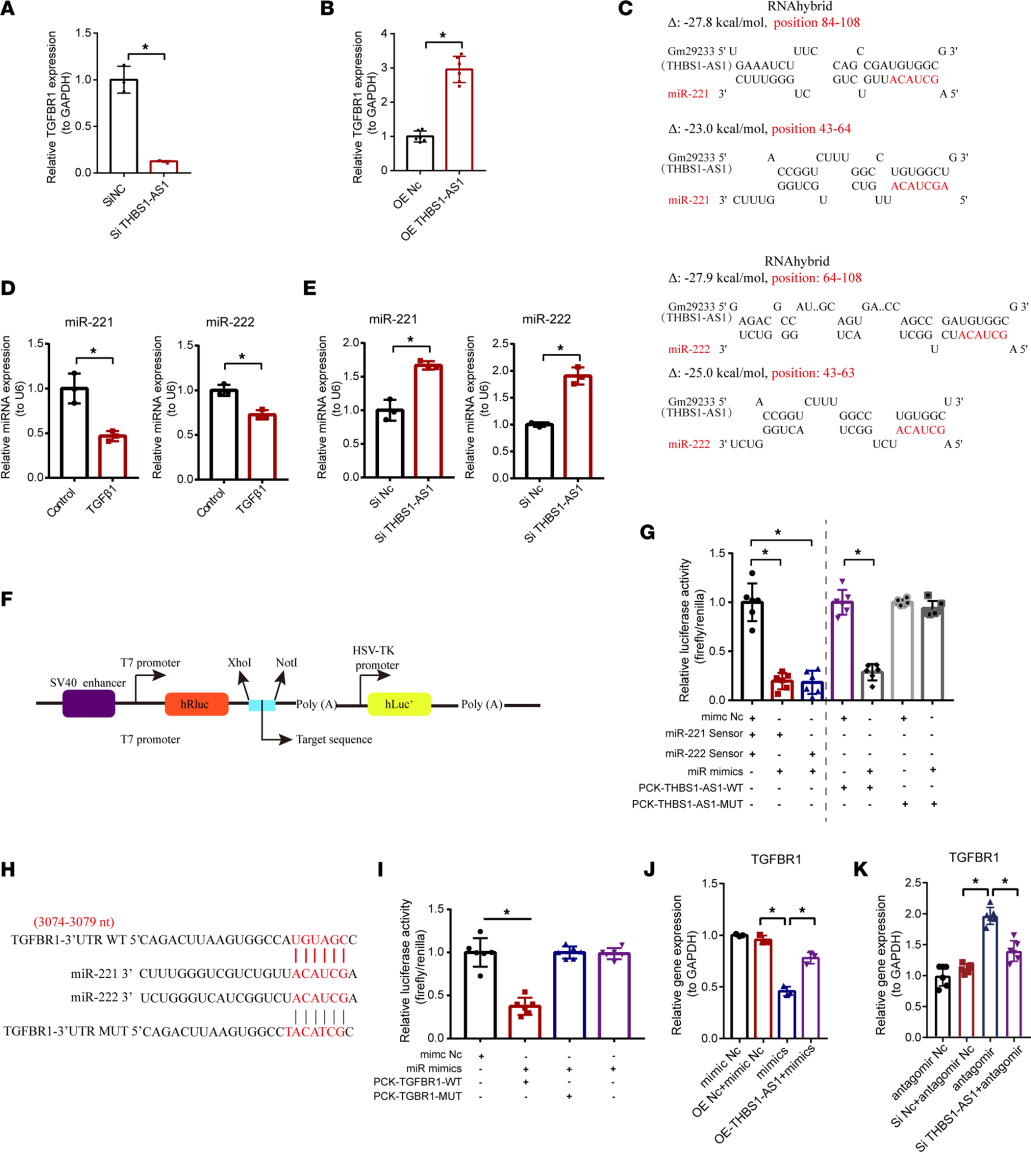
The identification of miR-221/222 as sponged targets of lncRNA THBS1-AS1 and TGFBR1. (**A**) The gene expression of TGFBR1 in cardiac fibroblasts after siRNA-mediated THBS1-AS1 knockdown by qPCR (*n* = 3). An unpaired, 2-tailed *t* test was used. (**B**) The gene expression of TGFBR1 in cardiac fibroblasts after adenovirus-mediated THBS1-AS1 overexpression by qPCR (*n* = 6). An unpaired, 2-tailed *t* test was used. (**C**) The predicted binding sites of THBS1-AS1 and miR-221/222. (**D**) The expression of miR-221 and miR-222 in cardiac fibroblasts after TGF-β1 treatment (*n* = 3). An unpaired, 2-tailed *t* test was used. (**E**) The expression of miR-221 and miR-222 in cardiac fibroblasts after THBS1-AS1 knockdown (*n* = 3). An unpaired, 2-tailed *t* test was used. (**F**) The schematic diagram of plasmid. (**G**) The verification of miR-221/222 as sponged targets of lncRNA THBS1-AS1 by the dual-luciferase reporter assay (*n* = 6). An unpaired, 2-tailed *t* test in the left panel and 1-way ANOVA followed by a Bonferroni post hoc test in the right panel were used. (**H**) The predicted binding sites of TGFBR1 and miR-221/222. (**I**) The verification of TGFBR1 as a target gene of miR-221/222 by the dual–luciferase reporter assay (*n* = 6). One-way ANOVA, followed by a Bonferroni post hoc test, was used. (**J**) The gene expression of TGFBR1 in cardiac fibroblast transfected with miR-221/222 mimics alone or transfected with miR-221/222 mimics and AAV-mediated THBS1-AS1 overexpression (*n* = 3). One-way ANOVA, followed by a Bonferroni post hoc test, was used. (**K**) The gene expression of TGFBR1 in cardiac fibroblast transfected with miR-221/222 antagomir alone or transfected with miR-221/222 antagomir and siRNA-mediated THBS1-AS1 knockdown (*n* = 6). One-way ANOVA, followed by a Bonferroni post hoc test, was used. The results are presented as means ± SEM; *P < 0.05.

As mentioned above, lncRNA THBS1-AS1 was found to be highly correlated with TGFBR1. Next, we validated this correlation in CFs. We observed that the mRNA expression level of TGFBR1 decreased by 87% after the siRNA-specific knockdown of lncRNA THBS1-AS1 (Figure 5A). In contrast, after the OE of lncRNA THBS1-AS1 using adenovirus, the gene expression level of TGFBR1 increased by 2-fold (Figure 5B).

miRNAs can be sponged by lncRNAs acting as their downstream regulators. A total of 37 target miRNAs for lncRNA THBS1-AS1 were predicted by RNAhybrid and miRanda software (Supplemental Figure 10, A and B). We found that, among them, miR–221-3p and miR–222-3p were able to interact with lncRNA THBS1-AS1 and each had 2 perfect complementary binding sites for the target sequences by RNAhybrid (Figure 5C). After TGF-β1 stimulation, the expression levels of miR-221 and miR-222 were decreased by 53% and 28%, respectively (Figure 5D), while, after the knockdown of lncRNA THBS1-AS1, the expression levels of miR-221 and miR-222 increased by 67% and 90%, respectively (Figure 5E). Furthermore, a dual luciferase reporter assay (Figure 5F) revealed that the OE of miR-221/222 significantly inhibited the luciferase activity of the miR-221/222 sensor compared with the mimic Nc group in the HEK293T cells (Figure 5G). In addition, the OE of miR-221/222 significantly reduced the activity of PCK–THBS1-AS1–WT, with no effect on the activity of PCK–THBS1-AS1–MUT (Figure 5G).

Then, we predicted that miR-221/222 was able to interact with TGFBR1 by RNAhybrid software. As shown in Figure 5H, 3074-3079 in the 3′UTR region of TGFBR1 could bind to miR-221 and miR-222. We constructed a luciferase expression vector carrying the targeting fragment containing the TGFBR1 binding sites or mutated sequences. The fluorescence activity of PCK-TGFBR1-WT decreased by approximately 61% when miR-221/222 was overexpressed, whereas the activity of PCK-TGFBR1-MUT did not change (Figure 5I). Furthermore, we found that the OE of the miR-221/222 group reduced the gene level of TGFBR1 in CFs compared with the mimic Nc group. Of further importance, in Figure 5J, compared with the control group, the TGFBR1 expression was downregulated after miR-221/222 OE, and the downregulation of TGFBR1 could be reversed by THBS1-AS1 OE. Also, the miR-221/222 knockdown upregulated TGFBR1 levels, and this trend could also be reversed by knocking down THBS1-AS1 (Figure 5K). All of these data suggest the dependence of THBS1-AS1 on miR-221/222 for regulating TGFBR1.

**Figure 6:**
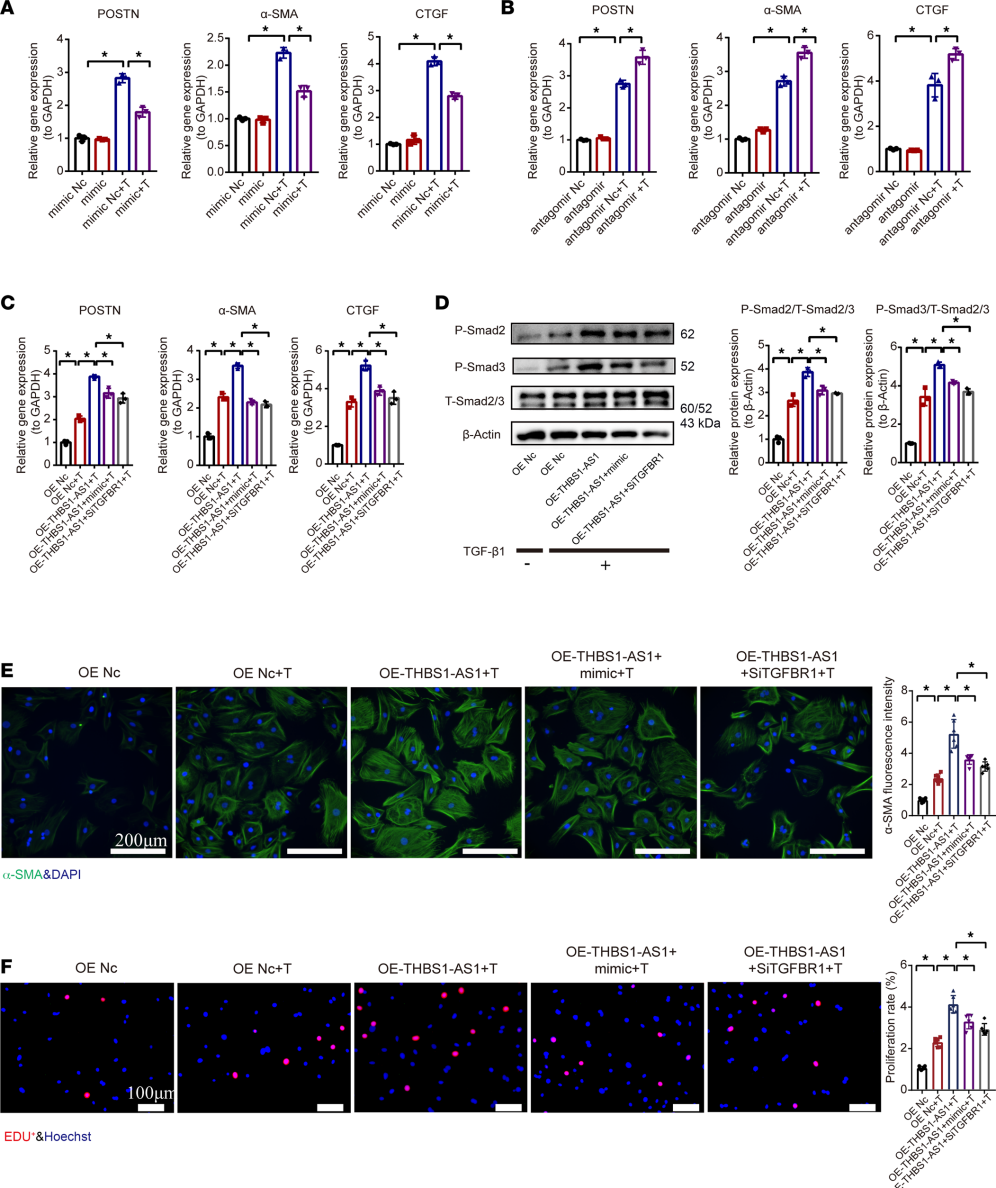
The regulation of cardiac fibroblast activation by lncRNA THBS1-AS1 via the miR-221/222-TGFBR1 axis. (**A** and **B**) The gene expression of CTGF, α-SMA, and POSTN in cardiac fibroblasts after miR-221/222 mimic or antagomir treatment with/without TGF-β1 stimulation (*n* = 3). (**C**) The gene expression of CTGF, α-SMA, and POSTN. (**D**) The protein expression of P-smad2, P-smad3, and T-smad2/3 (*n* = 3). (**E**) The α-SMA fluorescence intensity (*n* = 6). Scale bars: 200 μm. (**F**) The cell proliferation in cardiac fibroblasts transfected with adenovirus-mediated OE–THBS1-AS1 alone or miR-221/222 mimic and OE–THBS1-AS1 or siRNA-mediated TGFBR1 knockdown and OE–THBS1-AS1 treatment with/without TGF-β1 stimulation (*n* = 6). One-way ANOVA, followed by a Bonferroni post hoc test, was used. The results are presented as means ± SEM; *P < 0.05.

To further characterize the function of the THBS1-AS1–miR-221/222–TGFBR1 axis in cardiac fibrosis activation, we performed a series of loss- and gain-of-function experiments. We selected miR-221 and miR-222 mimics at concentrations of 50 nM to transfect primary mouse fibroblasts for 24 hours and found that the expression levels of miR-221/222 increased 229-fold and 312-fold, respectively (Supplemental Figure 11A). In addition, 100 nM specific miR-221/222 antagomir was able to downregulate miR-221 and miR-222 expression by nearly 66% and 65% (Supplemental Figure 11A). The OE of miR-221/222 with mimics reduced CF activation (Figure 6A), whereas the knockdown of miR-221/222 with antagomirs promoted TGF-β1–induced activation of CFs (Figure 6B). The silencing efficiency of all 3 siRNAs for TGFBR1 reached over 70%. Among them, we selected si-TGFBR1_1 for our further study due to its 80% silencing efficiency (Supplemental Figure 11B). To determine whether lncRNA THBS1-AS1 regulates CFs activation through the miR-221/222–TGFBR1 axis, (a) miR-221/222 antagomirs treatment after lncRNA THBS1-AS1 knockdown or (b) miR-221/222 mimics or si-TGFBR1 (the knockdown of the TGFBR1 expression) treatment after THBS1-AS1 OE was carried out. The results showed that, under TGF-β1 stimulation, the expression of fibrosis markers, the fluorescence intensity of α-SMA, proliferation, migration, and the collagen contraction of CFs were significantly aggravated in the OE of the THBS1-AS1 group, and the phenotype was reversed by miR-221/222 OE or the TGFBR1 knockdown group (Figure 6, C–F, and Supplemental Figure 12, A and B).

### Knockdown of lncRNA THBS1-AS1 specifically in CFs alleviated TAC-induced cardiac fibrosis in mice.

**Figure 7:**
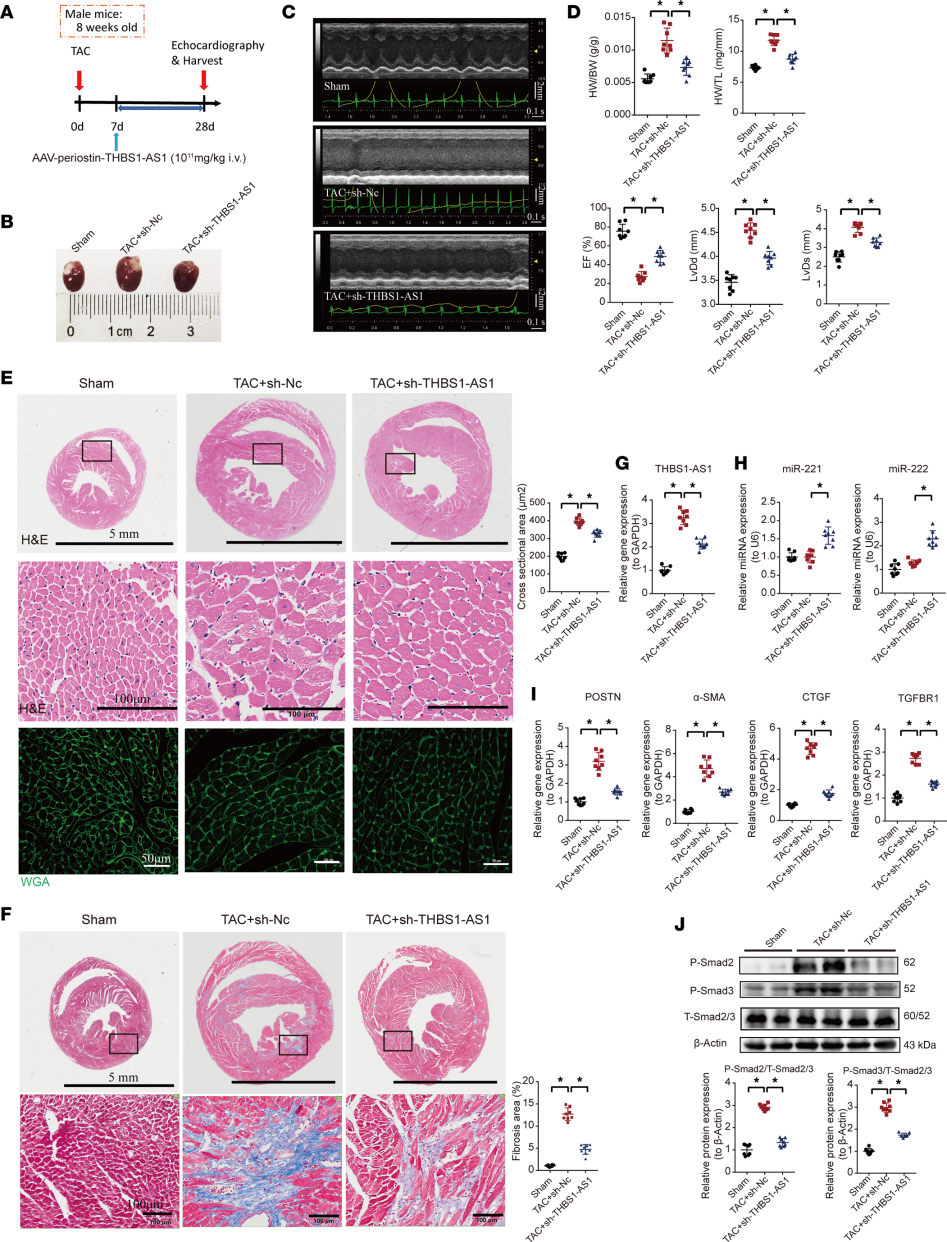
The specific knockdown of lncRNA THBS1-AS1 targeting activated cardiac fibroblasts alleviates TAC-induced cardiac fibrosis in mice. (**A**) The schematic diagram outlining the in vivo experiments. The mice were treated with AAV2/9–periostin promoter–shTHBS1-AS1 at 7 days after TAC. (**B**) The gross appearance of the hearts obtained from the mice. (**C**) The representative images of echocardiography from mice. (**D**) The cardiac function indicators measured by echocardiography and anatomy index (LVEF [%], LVIDd, LVIDs, and HW/BW) in mice. (**E**) The representative H&E images and WGA quantification of transverse-sections of mouse hearts. Scale bars: 5 mm (upper panels), 100 μm (middle panels), and 50 μm (lower panels). (**F**) The representative Masson trichrome staining images of transverse-sections of mouse hearts. Scale bars: 5 mm (upper panels) and 100 μm (lower panels). (**G**) The gene expression of lncRNA THBS1-AS1 in mouse hearts by qPCR. (**H**) The expression of miR-221 and miR-222 in mouse hearts by qPCR. (**I**) The gene expression of POSTN, CTGF, α-SMA, and TGFBR1 in mouse hearts by qPCR. (**J**) The protein expression of P-smad2, P-smad3, and T-smad2/3 in mouse hearts by Western blot assay (*n* = 8 in each group). One-way ANOVA, followed by a Bonferroni post hoc test, was used. The results are presented as means ± SEM; *P < 0.05.

To study the role of THBS1-AS1 in cardiac fibrosis in vivo, mice were treated with either (a) shRNA that contained periostin promoter, allowing the knockdown of THBS1-AS1 specifically in activated CFs, or (b) a Nc shRNA (shNc) by administering the adeno-associated virus (AAV) via tail vein 7 days after TAC (Figure 7A). Compared with the Sham group, mice in the TAC+shNc group developed significant cardiac fibrosis accompanied by a deterioration of cardiac function 4 weeks after TAC (Figure 7, B–F). As excepted, compared with the TAC+shNc group, the HW/BW ratio, EF, LVIDd, and LVIDs in the TAC+sh-THBS1-AS1 treatment group were significantly improved (Figure 7, B–D), and the average myocyte cross-sectional area and deposition of cardiac collagen fibers were also reduced (Figure 7, E and F). Moreover, qPCR and Western blot were used to evaluate the expression of fibrosis markers in the hearts. The expression level of lncRNA THBS1-AS1 was increased 2.3-fold in the TAC+shNc group compared with the Sham group (Figure 7G). Compared with the TAC+shNc group, the expression level of lncRNA THBS1-AS1 in the TAC+sh-THBS1-AS1 group was decreased by 34.5% (Figure 7G). The decrease in THBS1-AS1 levels was accompanied by increases in the expression levels of miR-221 and miR-222 by 58.5% and 80%, respectively (Figure 7H). All of these improvements were associated with the reduction in the levels of cardiac fibrosis markers, P-Smad2/T-Smad2/3 and P-Smad3/T-Smad2/3 (Figure 7, I and J). These observations indicate that the knockdown of lncRNA THBS1-AS1 specifically in CF alleviated the deterioration of the cardiac function and cardiac fibrosis caused by pressure overload in mice.

### Human THBS1-AS1 alleviated CF activation by regulating TGFBR1.

In order to find the potential translational value of lncRNA THBS1-AS1, we performed the conservativeness analysis of lncRNA THBS1-AS1 between human and mouse. We identified a positional conserved lncRNA THBS1-AS1 (ENSG00000278621) on the human genome, which is located on the negative strand of chromosome 15: 39588357-39588882, the antisense strand of THBS1, with a transcript of ENST00000616754.1 and a length of 526 bp (Supplemental Figure 13, A and B). MAFFT software identified the conserved position and the sequence conservativeness between the mouse THBS1-AS1 and the human THBS1-AS1 (Supplemental Figure 13C). In addition, the RNA secondary structure of the human and mouse THBS1-AS1 was predicted by RNA fold (http://rna.tbi.univie.ac.at/cgi-bin/RNAWebSuite/RNAfold.cgi) and visualized by Forna (http://rna.tbi.univie.ac.at/forna/). The results showed that the human and mouse THBS1-AS1 had some similarities (Supplemental Figure 13D).

**Figure 8:**
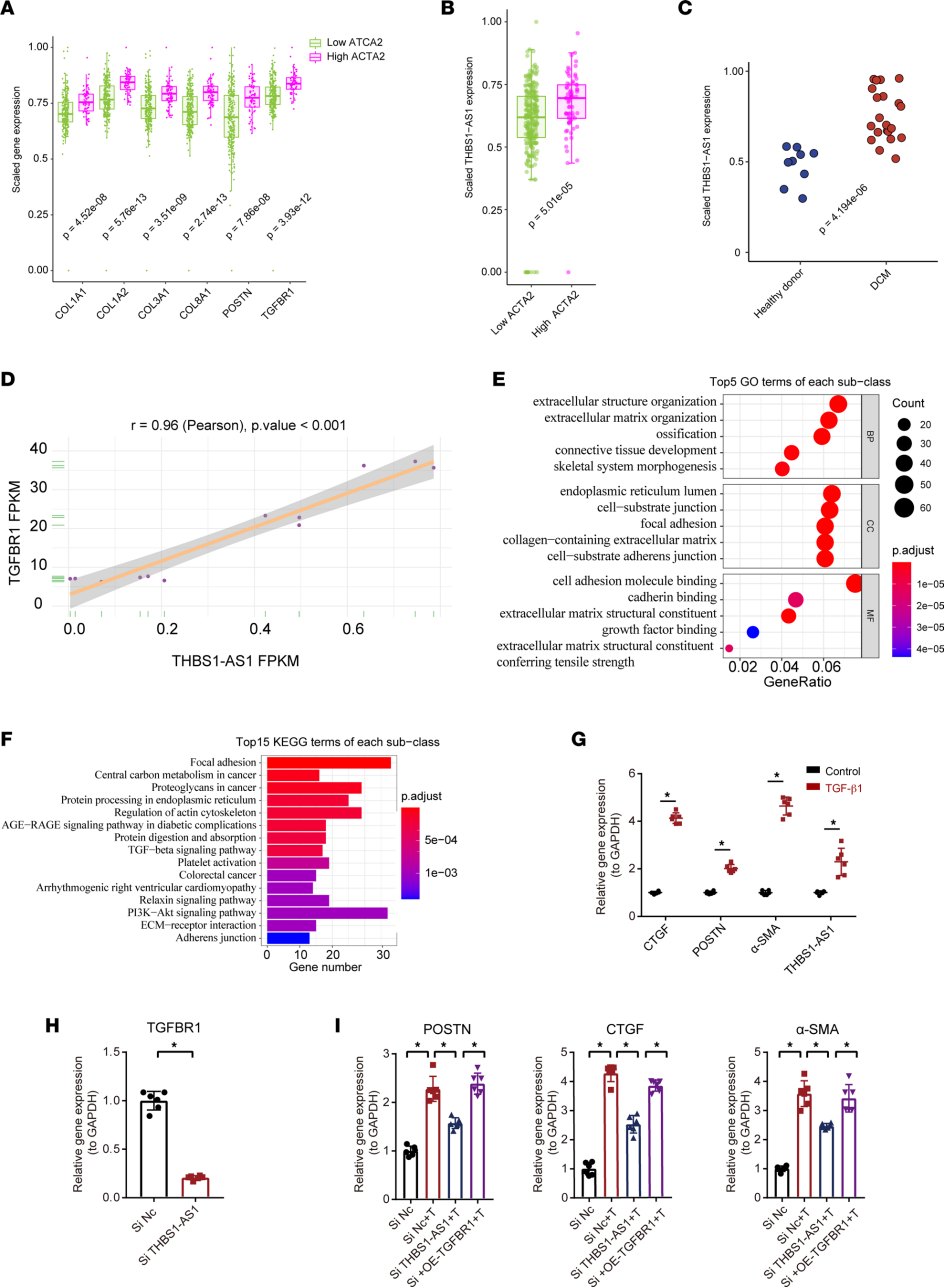
The role of THBS1-AS1 on TGFBR1 in human activated cardiac fibroblasts. (**A** and **B**) GTEx bulk RNA-Seq data, expression of markers of cardiac fibrosis, and THBS1-AS1 in human hearts. The gene expression levels (TPM) are graphed normalized to their maxima. A 2-sided Wilcoxon rank-sum test was used. (**C**) The bulk RNA-Seq data of human THBS1-AS1 expression in heart tissue in healthy donors and patients with dilated cardiomyopathy (DCM) (GSE135055). The gene expression levels (TPM) are graphed normalized to their maxima. A 2-sided Wilcoxon rank-sum test was used. (**D**) The correlation between THBS1-AS1 and TGFBR1 analyzed by the Pearson’s correlation test (GSE152250). (**E** and **F**) The rank-based GO analysis (BP, CC, and MF) and the KEGG pathway enrichment analysis of genes significantly associated with THBS1-AS1 identifies the top 15 enriched pathways. (**G**) The gene expression of CTGF, POSTN, α-SMA, and THBS1-AS1 in human cardiac fibroblasts stimulated with TGF-β1 for 24 hours by qPCR (*n* = 6). An unpaired, 2-tailed *t* test was used. (**H**) The gene expression of TGFBR1 in cardiac fibroblasts after siRNA-mediated THBS1-AS1 knockdown by qPCR (*n* = 6). An unpaired, 2-tailed *t* test was used. (**I**) The gene expression of CTGF, α-SMA, and POSTN in cardiac fibroblasts after interfering with siRNA-mediated THBS1-AS1 knockdown alone or interfering with THBS1-AS1 knockdown and plasmid-mediated TGFBR1 overexpression at the same time with TGF-β1 stimulation (*n* = 6). One-way ANOVA, followed by a Bonferroni post hoc test, was used. *P < 0.05. DCM, dilated cardiomyopathy. GO, Gene Ontology; BP, biological process; MF, molecular function; CC, cellular component; KEGG, Kyoto Encyclopedia of Genes and Genomes.

To determine the expression pattern of THBS1-AS1 in the context of human pathological cardiac remodeling, we queried bulk RNA-Seq of human heart tissues from the Genotype-Tissue Expression (GTEx) project. According to Acta2 (α-SMA), the marker of CF activation, we divided the population into higher Acta2 (top 20%) and lower Acta2 groups (Figure 8B). Consistent with the above results, THBS1-AS1 was significantly upregulated in the higher Acta2 group, accompanied by higher POSTN, collagen, and THSBR1 expression (Figure 8, A and B). Besides, the human THBS1-AS1 expression was upregulated in the heart tissue of patients with dilated cardiomyopathy (DCM) compared with healthy donors (Figure 8C).

The raw data of GSE152250 was used to further investigate whether THBS1-AS1 is involved in the process of cardiac fibrosis. In this database, researchers performed RNA-Seq of the human primary CFs 24 and 48 hours after stimulation with 5 ng/mL of TGF-β1 to induce myofibroblast activation (19). The cluster analysis identified treatment with good cluster separation (control, TGF-β1_24h, TGF-β1_48h) (Supplemental Figure 14A). Through our analysis, we found that, compared with the control group, the expressions of THBS1-AS1 and TGFBR1 were significantly upregulated in HCFs after TGF-β1 stimulation, and the upward increase was more significant after 48 hours compared with 24 hours (Supplemental Figure 14, B and C). THBS1-AS1 was positively correlated with TGFBR1, with a correlation coefficient of 0.96 (Figure 8D). Furthermore, through the functional annotation and pathway-gene coexpression network, THBS1-AS1 was significantly correlated with POSTN, CTGF (CCN2), α-SMA, and FBN1 and might be involved in the formation of the extracellular structure, the ECM formation, the connective tissue development, and the bone formation pathways (Figure 8, E and F, and Supplemental Figure 14D).

Furthermore, we used HCFs to further verify the expression of THBS1-AS1 in human activated CFs. As expected, the expression of THBS1-AS1 was upregulated in the activated HCFs induced by TGF-β1 compared with the control group (Figure 8G). In order to examine the role and regulatory control of THBS1-AS1 on TGFBR1 in human cells, we examined the effect of siRNA-mediated THBS1-AS1 knockdown on the TGF-β1–induced HCF activation. As expected, the expression of TGFBR1 was downregulated by si-THBS1-AS1 (Figure 8H). Also, fibrosis markers, POSTN, CTGF, and α-SMA were suppressed by the THBS1-AS1 knockdown in activated HCFs (Figure 8I). More importantly, the OE of TGFBR1 can restore the remission of activated CFs induced by THBS1-AS1 knockdown (Figure 8I and Supplemental Figure 15). These findings indicate that THBS1-AS1 regulates the activation of HCFs depending on TGFBR1. Moreover, to verify the role of THBS1-AS1 on activated CFs, human and mouse CFs were preconditioned with TGF-β1 for 24 hours prior to the knockdown of THBS1-AS1 treatment to observe the effect of THBS1-AS1 on activated fibroblasts. Consistent with silencing of THBS1-AS1 prior to fibroblast activation, THBS1-AS1 inhibition also alleviated the expression of fibrogenic genes after fibroblast activation (Supplemental Figure 16). Collectively, these observations suggest that THBS1-AS1 promotes CFs activation by regulating TGFBR1, and the modulation of THBS1-AS1 may have both preventive and therapeutic effects.

## Discussion

**Figure 9:**
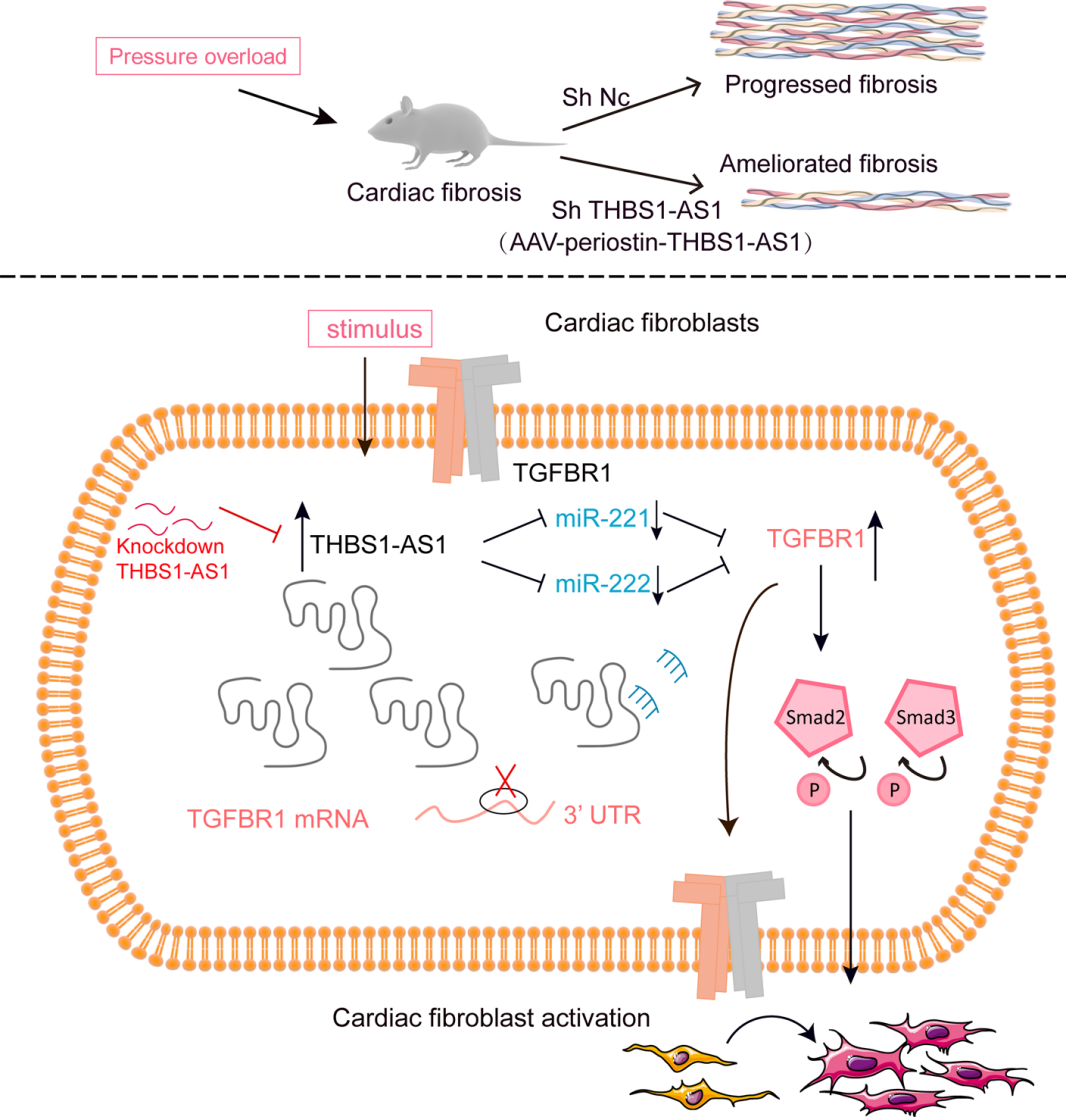
A diagram outlining the mechanism of lncRNA THBS1-AS1 in cardiac fibrosis. Under stress conditions, such as pressure overload and TGF-β1 stimulation, upregulated lncRNA THBS1-AS1 serves as a miRNA sponge, competitively binding to miR-221/222, inhibiting their effect on the targeted TGFBR1, and facilitating the phosphorylation of Smad2/3. The inhibition of THBS1-AS1 cardiac fibroblasts primed with TGF-β1 reduces fibrotic ECM gene expression and cardiac fibroblast activation. Moreover, cardiac fibroblast–specific knockdown of THBS1-AS1 improves cardiac function and reduces myocardial fibrosis in response to pressure overload. P, phosphorylation; UTR, untranslated regions; si, siRNA; sh, shRNA; Nc, negative control.

In the present study, we identified that lncRNA THBS1-AS1 was involved in the process of pressure overload–induced cardiac fibrosis, and the knockdown of lncRNA THBS1-AS1 alleviated CF activation in vitro and cardiac fibrosis in vivo. Mechanistically, lncRNA THBS1-AS1 regulated cardiac fibrosis through the miR-221/222–TGFBR1 axis (Figure 9). The implications of these findings are discussed below.

Several recent studies provided strong evidence that lncRNAs, such as Cfast, Dnm3os, PFL, PCFL, and NEAT1, play essential roles in cardiac fibrosis (20–24). Sun et al. demonstrated that PCFL could negatively regulate miR-378, which in turn regulates GRB2, thus playing a promotional role in cardiac fibrosis induced by myocardial infarction (20). In a mouse model of myocardial infarction, Zhang et al. found that lncRNA Cfast exacerbates cardiac fibrosis by competitively inhibiting the COTL1 interaction with TRAP1, a fibrogenic factor (22). LncRNA Neat1 promotes cardiac fibrosis by recruiting EZH2 to suppress Smad7, which leads to the activation of p-Smad2/3 (24). Previous studies have found that, in patients with proliferative vitreoretinopathy, THBS1-AS1 is associated with the THBS1 gene (25), a profibrotic molecule (26). Also, THBS1-AS1 is upregulated in longevous elders compared with normal elders, and its OE decelerates cellular senescence (27). In this study, using an unbiased bioinformatic approach followed by biological validations, we screened lncRNA THBS1-AS1 and characterized its profibrotic role in the process of pressure overload and TGF-β1–induced cardiac fibrogenesis. It is noteworthy to mention that the THBS1-AS1 knockdown demonstrated not only a preventive role, but also therapeutic effects against the cardiac fibrosis, as evidenced by diminished levels of fibrotic markers and the improvement of cardiac function prior to or after fibroblast activation.

We also investigate the mechanism of THBS1-AS1 in regulating fibroblasts activation. The GSEA further illustrated the profibrotic function of THBS1-AS1 associated with TGF-β signaling. We focus on TGFBR1, a therapeutic target in the field of cardiac fibrosis. TGF-β has been reported to bind heterodimeric receptors consisting of TGF-β receptors 1 and 2 (TGFBR1/2) in the plasma membrane, thereby inducing phosphorylation of Smad2 and Smad3 transcription factors (28, 29). This is the classical pathway that activates fibroblast differentiation to myofibroblasts and promotes ECM production (29). The specific knockdown of Smad2/3 in activated fibroblasts can reduce the extent of cardiac fibrosis after pressure overload but cannot reverse myocardial hypertrophy and heart failure, whereas the specific knockdown of TGFBR1/2 can alleviate both cardiac fibrosis and hypertrophy (30). Therefore, TGFBR1/2 has become one of the most interesting therapeutic targets in the field of cardiac fibrosis. However, since TGF-β signaling is involved in cellular differentiation and intratissue homeostasis, extensive inhibition of TGF-β signaling may lead to serious side effects, including liver toxicity (31) and cardiotoxicity (11, 12). A deficiency of TGFBR1 (global deletion) in mice is lethal in their embryonic stages (32). A conditional deletion of TGFBR1 in the lung epithelium or mesenchyme also leads to abnormal lung development (33). Therefore, directly targeting TGF-β or TGF-β receptors may not be a viable option for treating cardiac fibrosis. Instead, intervening with endogenous ncRNAs targeting TGF-β signaling may be an alternative option for the treatment of fibrosis (34, 35). LncRNA Neat1 activates TGF-β signaling by inhibiting Smad7 expression, while AAV9-mediated Neat1 knockdown alleviates TAC-induced cardiac fibrosis (24). Interestingly, our further studies have demonstrated that the promotion effect of THBS1-AS1 on CFs activation can be reversed by TGFBR1 knockdown. These findings indicate that THBS1-AS1 regulates cardiac fibrosis depending on TGFBR1 in activated CFs.

Emerging studies demonstrate that lncRNA competitively binds to miRNA to regulate the expression of target mRNA. In addition, miRNA also plays an important role in regulating the progression of cardiac fibrosis by targeting the TGF-β signaling pathway (36). miR–221-3p and miR–222-3p (miR-221/222) encode in the same genomic region and exhibit a high degree of sequence similarity (37, 38). Zhou et al. observed a role of miR-221 in a model of severe renal failure–induced cardiac fibrosis and found that miR-221 exerted antifibrotic effects by directly targeting TSP1 and improved the deterioration of the left ventricular function (39). Furthermore, in patients with systolic or diastolic dysfunction, cardiac interstitial fibrosis was associated with low miR-221/222 levels; in a mouse model of angiotensin II–induced cardiac remodeling and heart failure, the inhibition of miR-221/222 was able to promote cardiac interstitial fibrosis, cardiac dilation, and dysfunction (37). In line with these findings, we show that miR-221/222 levels were significantly downregulated during CF activation. However, we do not see endogenous changes of these miR-221/222 in heart tissues of the TAC model. The reason may be that the expression of miR-221/222 in CF and cardiomyocytes are different in the process of cardiac remolding. Consistent with our results, Verjans et al. found that, in activated CFs, TGF-β1 mediated the downregulation of miR-221/222 (37). In cardiac hypertrophy induced by exercise, miR-222 is induced in cardiomyocytes but not in noncardiomyocytes (40). Interestingly, we also found that miR-221/222 played a protective role in the activation of CFs. Further studies verified the direct binding relationship between miR-221/222 and TGFBR1/THBS1-AS1. It is worth noting that miR-221/222 knockdown upregulated TGFBR1 levels, and this trend could also be reversed by the knockdown of THBS1-AS1 in CFs. In vivo, the specific knockdown of lncRNA THBS1-AS1 in activated CFs significantly downregulated the TGFBR1 expression and upregulated miR-221/222 during TAC-induced cardiac fibrosis. These pieces of evidence suggest that THBS1-AS1 promotes TGFBR1 expression by binding to miR-221/222. There is an important point we need to pay attention to; currently, most studies have explored the competitive effect of lncRNA and mRNA on miRNA, but few studies have investigated whether there is a dose-effect relationship between lncRNA/mRNA and miRNA, which may be the cause of the contradiction in some studies. Similarly, our results showed that knockdown of THBS1-AS1 could restore the elevated TGFBR1 expression induced by miR-221/222 knockdown. However, we did not explore the dose-effect relationship between THBS1-AS1/TGFBR1 and miR-221/222; this relationship is worthy of further exploration in future studies, both at the posttranscriptional and posttranslational levels.

Most lncRNAs evolve rapidly. However, only some sequences are multispecies conserved, which is closely related to their function (41). At present, a comparative analysis of lncRNA’s conservation and function between species has not been carried out in most cases. Most lncRNAs are poorly conserved between mice and humans, which is mainly reflected in the sequence similarity and length of lncRNAs. Unfortunately, although there are some similarities in the secondary structure, putative binding sites for miR-221/222 were not present in the human homologs of THBS1-AS1 in our study. However, the poor conservation of lncRNAs does not mean a lack of functional conservation. The very poorly conserved lncRNA X-inactive specific transcript (Xist) demonstrated that it plays a key role in the regulation of imprinting and random X inactivation among species (42, 43). In our study, although the sequence of THBS1-AS1 is poorly conserved, we demonstrated that it can also affect the activation of HCFs by regulating TGFBR1, consistent with the results in the mice experiment. The observations that fibrosis markers were suppressed by the THBS1-AS1 knockdown in activated HCFs and that the OE of TGFBR1 can restore the remission of activated CFs induced by the THBS1-AS1 knockdown suggest a functional conservation of THBS1-AS1 among species. In addition, lncRNA can regulate some important molecules through a variety of pathways. Interestingly, lncRNA NEAT1 can regulate the Wnt signaling pathway via multiple routes. NEAT1 knockdown blocked Wnt signaling via miR-34a in colorectal cancer progression (44). Also, NEAT1 activates Wnt signaling by interacting with DDX5 to promote colorectal cancer progression (45). Therefore, future studies are needed to further investigate the mechanism of how THBS1-AS1 regulates TGFBR1 in the process of human fibroblasts activation.

There are several limitations in this work. First, although the inhibition of THBS1-AS1 alleviated CFs’ activation by modulating TGF-β signaling, other cells like macrophages may secrete TGF-β to mediate fibroblast activation in the process of cardiac fibrosis. In our study, we partially alleviated fibrosis by depriving the profibrotic role mediated by THBS1-AS1 elevation in CFs. Through a single-cell sequencing analysis (GSE120064), we found that the expression of TGFB1 in macrophages was much lower than that in CFs in the Sham and TAC mice (Supplemental Figure 17). In addition, compared with CF, the expression level of THBS1-AS1 in macrophages is very low and does not increase significantly even after TGF-β1/LPS stimulation (Supplemental Figure 18). These observations suggest that the activation of THBS1-AS1–mediated TGF-β signaling in CFs but not macrophages contribute to the cardiac fibrosis and suggest that targeting this signaling in CFs is effective for the treatment. However, the potential crosstalk between macrophages and CFs for the development of cardiac fibrosis deserves further investigation. Second, several immune cells including macrophages express periostin, which may interfere with the specificity of the AAV2/9–periostin promoter when a loss of function for THBS1-AS1 in fibroblasts was applied in mice in vivo. However, the expression of POSTN in these cells, including macrophages, was much lower than that of CFs, evidenced by using the single-nucleus sequencing data from the myocardium of patients with DCM (46). Third, siRNA-mediated THBS1-AS1 knockdown may not be optimal because of incomplete and potential off-target effects. Other knocking-down methods, including CRISPR/CAS9 approaches, can be more efficient. However, the generation of a CF immortalized cell line was attempted but was found to be difficult. In addition, primary CFs were inappropriate for a long-term culture in vitro due to self-activation properties. To avoid off-target effects and to offer more rigorous evidence, additional experiments in the present study using ASO confirmed an efficient knockdown of THBS1-AS1 in activated CFs. Future studies using CF-specific THBS1-AS1–KO mice will increase insights in understanding the mechanisms of THBS1-AS1–mediated cardiac fibrosis. Fourth, the TAC-induced cardiac fibrosis does not completely represent the complex conditions of clinical heart failure. In most patients of heart failure, a variety of conditions, such as hypertension, type 2 diabetes, and hyperlipidemia are very common and coexisting. Therefore, different models of heart failure, such as diabetic cardiomyopathy and myocardial infarction, combined with other diseases are required to validate the clinical effect of THBS1-AS1 treatment. Fifth, our data suggest the possibility that THBS1-AS1 inhibition exerts both preventive and therapeutic effects on the heart during the development and progression of cardiac fibrosis. However, more clinical relevance of silencing approach targeting THBS1-AS1 remains to be tested before and after development of cardiac fibrosis. Also, as we used noninvasive echocardiography to assess cardiac function, the carotid arteries were not cannulated for pressure monitoring. The magnitude of trans-TAC pressure gradient fluctuations needs to be further evaluated in future investigations. Finally, in view of the nonphysiologic levels of miRNA and lncRNA to establish this interaction, some in vivo experiments are more reflective of physiological conditions, such as RNA immunoprecipitation assay, biotin-coupled probe pull-down assay, and they should also be considered systematically in future studies.

In conclusion, our work reveals that a newly identified lncRNA THBS1-AS1 drives CF activation and fibrosis induced by pressure overload via mechanisms involving miR-221/222 sponging and TGFBR1 activation. The lncRNA THBS1-AS1 might have translational values and may serve as a target for the treatment of cardiac fibrosis.

## Methods

### Animals and TAC surgery.

The C57BL/6 male mice (6–8 weeks, 20–22 g at the time of the experiments) used in this study were purchased from Beijing Weitonglihua animal Technology Co. (Animal Production License No. SCXK [Beijing] 2016-0006). The animals were housed in a specific pathogen–free (SPF) environment with free access to water and autoclaved standard chow in the animal experiment center of West China Second Hospital (Sichuan, China). The TAC operation was performed on mice to induce cardiac fibrosis as we previously reported (47). Briefly, mice were anesthetized by inhalation with a mixture of isoflurane (2% induction, 0.5% maintenance) and oxygen (100%, 0.5–1.5 L/min). The anesthetized mice were intubated with a PE 90 tubing in a supine position on a heated operating table. After successful intubation, the trachea was connected to a small animal ventilator for mechanical ventilation. Under the operation microscope, the aortic arch was exposed and constricted with a 27-gauge blunt needle (silk thread). After constriction, the needle was removed. The Sham-operated group underwent the same operation without ligation. Two, 4, and 8 weeks after TAC, the mouse underwent echocardiography. At the end of the experiment, the mouse was euthanized by CO_2_ inhalation according to the AVMA Guidelines (https://www.avma.org/resources-tools/avma-policies/avma-guidelines-euthanasia-animals). The hearts of the mice were immediately snap-frozen in liquid nitrogen for transport and stored at −80°C for subsequent analysis. Blood samples taken from the retro-orbital sinus were analyzed using an automatic biochemical parameter analyzer to evaluate the liver/kidney function.

### Echocardiography.

Animals were anesthetized with 2% isoflurane inhalation, with the heart rate holding between 450 and 500 beats/min. Ultrasound imaging was performed using Vevo 3100 echocardiography (VisualSonics Inc.) to evaluate cardiac function. Aortic arch plane images were acquired in Pulse-wave Doppler Mode and Color Doppler Mode. M-mode images were obtained after the papillary muscle was clearly observed in the short-axis view (Supplemental Figure 1, A and B). Vevo lab 3.2.6 analysis software was used to process the acquired images and to calculate the peak aortic flow velocity, LVIDd, LVIDs, IVSd, LVPWd, and left ventricular EF.

### RNA-Seq.

The left ventricular tissues of mice were collected from the Sham, TAC_2w, and TAC_4w groups. Total RNA was extracted from the left ventricle of the mice using Trizol (Thermo Fisher Scientific). RNA samples were then sequenced as described in other studies (48, 49). Briefly, Strand-specific libraries were generated by the NEBNext Strand-Specific Library Construction Kit (NEB) according to the instructions of the manufacturer. Sequencing was performed using Illumina’s NovaSeq platform according to the manufacturer’s instructions. The paired-end clean reads were mapped to the mouse genome (mm10) using the HISAT2 software (50). The genes were annotated using the Ensemble database. The expression of both lncRNAs and coding RNAs was quantified using Cuffdiff (v2.1.1) (51). We have uploaded the RNA-Seq data to a public data set, SAR (accession PRJNA: 787574).

### Bioinformatic analysis.

The genes with FDR < 0.05 and log_2_ (fold change) > 1 were considered differentially expressed genes (DEGs) identified by edgeR (52). A gene ontology (GO), a KEGG, and a Reactome analysis were performed using the clusterProfiler (53).

WGCNA constructs a scale-free network by correlating gene expression with external traits, which is helpful for identifying candidate biomarkers or therapeutic targets. We performed the analysis using the WGCNA package (54) as follows. (a) Quality control. Fifteen samples with a total of 9,170 lncRNAs were included in the study. The exclusion criteria were samples with very low gene expression values or too many missing values. After quality control, all of the samples were qualified. The adjacency function was used to calculate the sample correlations, and samples with correlations less than –0.6 were excluded. Finally, 15 samples with 4,370 lncRNAs were included for further analysis. (b) Module clustering. According to the standard scale-free networks, we chose a soft threshold, 9, to construct the adjacency matrix. Afterward, the adjacency matrix was transformed into a topological overlap matrix (TOM). The minimum cutoff of genes per module was set to 30 and height cutoff to 0.25. Based on the TOM matrix, the hierarchical clustering method was performed to cluster genes into modules. (c) Finding the module and genes of interest. The included traits were stage (normal, cardiac hypertrophy, and heart failure), alanine transaminase (ALT), aspartate transaminase (AST), total bilirubin (TBIL), serum creatine (Cr), body weight, EF, and HW/BW ratio. A Pearson correlation analysis was performed to calculate the correlation between the modules and the phenotypes. Genes with high connectivity in the module of interest were selected as our hub genes for further analysis.

A GSEA was performed to explore the function of lncRNA THBS1-AS1. Based on our sequencing data, the correlation coefficients of lncRNA THBS1-AS1 with all of the mRNAs were calculated and ranked. The Molecular Signatures Database (MSigDB) was used as a reference to calculate the Enrichment Score (ES) for each gene set using the R package Pi (55). Based on the ES, the FDR value was obtained using the permutation test. Pathways with FDR < 0.05 were considered significantly enriched.

### Histological analyses using Masson’s trichrome staining, WGA, and H&E staining.

The heart tissues were routinely dehydrated, paraffin-embedded, and sectioned at 5 μm for H&E staining. H&E staining, Masson’s trichrome staining, and WGA were performed as previously described (56). To evaluate the degree of cardiac fibrosis, the ratio of blue-stained (collagen) to the total tissue area was assessed using ImageJ software (NIH; 10–12 sections were randomly selected per heart).

### Isolation and culture of CFs from neonatal mice.

Neonatal mouse CFs were isolated from 1- to 3-day-old C57BL/6 mice. Briefly, the dissected hearts were washed with ice-cold PBS and cut into small pieces (0.5–1 mm^3^). Then the hearts were dissociated using 0.25% trypsin (Sigma-Aldrich) placed on a horizontal shaker at 4°C, 20–30 rpm, overnight. Pooled cell suspensions were resuspended in digestion buffer and 1.5 mg/mL collagenase (type II collagenase; Sigma-Aldrich) at 37°C, 20–30 rpm, for 30 minutes. The solution of tissue was gently disassociated by pipetting and passed through a 70 μm cell strainer. Finally, the collected cells were centrifuged (37°C, 200g, for 5 minutes), resuspended, and differentially plated for 40 minutes in a DMEM supplemented with 10% fetal bovine serum (FBS) and penicillin-streptomycin (100 U/mL) (all from Thermo Fisher Scientific). Adherent CFs were cultured after 2 generations for further experiments. CFs at the second passaging were added into 12-well plates and cultivated to reach 70%–80% confluence. The CFs were starved for 4 hours with a serum-free medium; they were then later administered mouse TGF-β1 (10 ng/mL, Sino Biological Inc.) for 24 hours to induce fibroblasts activation.

### Culture of HCFs.

HCFs were purchased from ScienCell (catalog 6330) and maintained according to the manufacturer’s protocols. Second to third passages of HCFs were grown in 12-well plates until 70%–80% confluency. After starving for 4 hours with a serum-free medium, HCFs were treated with human TGF-β1 (10 ng/mL, Sino Biological Inc.) for 24 hours to induce fibroblasts activation.

### Immunofluorescence methodology.

Immunofluorescence staining of CFs was performed as described previously (47). The primary antibody for incubation was anti–α-SMA (Abcam, ab124964, 1:200). Images obtained from Confocal microscopy were analyzed with ImageJ software (NIH).

### In vitro scratch assay.

To verify the migration of CFs, we performed scratch assays as previously described (57). Passage 1 CFs were seeded onto 6-well plates. When the culture reached subconfluence, a scratch was then made using a standard 200 μL pipette tip. Cells were immediately washed twice with PBS and replaced with culture media with 1% FBS. To monitor the wound closure, photographs were taken at the same location at indicated time points after scratch. The scratch area was analyzed and counted by ImageJ software.

### Collagen contraction.

A contraction assay was performed according to the manufacturer’s manual. CFs (1 × 10^5^ cells/mL) were mixed with 5 mg/mL neutralized rat tail collagen solution (85% 5 mg/mL rat tail collagen type I with 5% 0.1 mol/L NaOH and 10% 10× PBS). Fibroblast-containing collagen solutions were plated at a density of 1 × 10^4^/well in a 24-well plate at 37°C for 30 minutes. Then, a cell culture medium mixed with treatment was added to the plate and incubated for 2 hours. Following incubation, the gel was released from the edge with a pipette tip. After 72 hours, the percentage of gel contraction was recorded and evaluated using ImageJ software.

### EdU assay.

The cell proliferation ability of CFs was carried out using a Click-iT EdU Cell Proliferation Assay (Click-iT EdU Alexa Fluor 555 Imaging Kit, Thermo Fisher Scientific). The cells were stained by incubating them with 50 μM EdU for 1 hour, and the cell nuclei were stained with 5 μg/mL DAPI for 30 minutes. The EdU^+^ rate was defined as the averaged ratio of EdU^+^ nuclei to the total number nuclei (%). All assays were performed 3 or more times.

### siRNA/ASO transfection.

ASO against THBS1-AS1, siRNA targeting THBS1-AS1, TGFBR1, and MEOX1 were synthesized by RiboBio. The target sequences were provided in Supplemental Table 1. Targeting siRNAs/ASO and scramble were transfected into CFs for 24 hours using Lipofectamine RNAiMAX Reagent (Thermo Fisher Scientific).

### Plasmid transfection.

TGFBR1 plasmid was synthesized by HonorGene Co. HCFs were transfected with pcDNA3.1-TGFBR1 or empty plasmid (1 μg/μL) using Lipofectamine 3000 Transfection Reagent (Invitrogen) for 24 hours.

### Adenovirus transduction.

THBS1-AS1 adenoviruses were constructed and purchased from Hanheng Biotechnology using the AdEasy vector. For THBS1-AS1 OE, CFs were infected with Ad-THBS1-AS1 or Ad-ctr (multiplicity of infection 300) as previously described (57).

### Prediction, transfection, and measurement of miRNA.

RNAhybrid (58) and miRanda (59) were applied to analyze the potential miRNA targeting sites within 3′UTR and lncRNA. When the cell density reaches 50%–60%, miRNA mimic Nc (50 nM), miRNA mimics (50 nM), miRNA antagomir Nc (100 nM), and miRNA antagomir (100 nM) were transfected by Lipofectamine 3000 (Invitrogen). For miRNA, cDNA was synthesized by using the All-in-OneTM miRNA qPCR Detection Kit (GeneCopoeia). Mmu-miR–221-3p primer (MmiRQP0338), mmu-miR–222-3p (MmiRQP0339), and snRNA U6 qPCR primer (MmiRQP9002) were purchased from Genecopoeia. The qPCR program was conducted in the BIO-RADCFX96 Real-Time PCR Detection System, and U6 was served as the reference gene.

### Dual-luciferase reporter assay.

The fragment of the THBS1-AS1 containing conserved miR-221/222 binding sites or mutated sequences was amplified and cloned into the psiCHECK-2 vector (Promega) at the 3′ end of the Renilla gene using primers containing Not I or Xho I sites (TsingKe Biotech) and T4 DNA ligase. Consistently, partial 3′UTR fragments of the TGFBR1 coding region containing the indicated miR-221/222 binding, or mutated sites were PCR amplified and cloned into the vector (psi-CHECK2-TGFBR1-W/Mut). All primer sequences are shown in Supplemental Table 3. A miR-221/222 sensor was designed by inserting miR-221/222 complementary sequences into the psiCHECK-2 vector. Next, 293T cells were cotransfected with miR-221/222 mimics and luciferase reporter plasmid (PCK–THBS1-AS1–WT/MUT or PCK–TGFBR1–WT/MUT). After transfecting for 48 hours, activities of firefly luciferase (FL) and Renilla luciferase (RL) were measured using the Dual-Luciferase Reporter Assay System (Promega).

### Nucleus-cytoplasm RNA separation assay.

According to the manufacturer’s manual, the assay was performed using Nuclear and Cytoplasmic Extraction Reagents (Thermo Fisher Scientific). The nuclear and cytoplasmic RNA fractions were extracted for reverse transcription PCR (RT-PCR) to detect the subcellular localization of THBS1-AS1. U6 and GAPDH were used as internal reference genes of the nucleus and cytoplasm, respectively.

### FISH.

RNA-FISH was used to detect the localization of lncRNA THBS1-AS1 in CFs. The hybridization of the target THBS1-AS1 and the nucleotide probe can be formed through denaturation, annealing, and renaturation. The FISH probe with a Cy3-specific effect on THBS1-AS1 was designed and synthesized by GenePharma (Shanghai GenePharma), and the specific sequence of the probe is shown in Supplemental Table 2. The cells were immobilized, prehybridized with hybridization solutions, and incubated with the THBS1-AS1 probes according to the manufacturer’s instructions. Finally, fluorescence images were taken using confocal microscopy (Olympus).

### AAV construction and infection.

AAV2/9 carrying a periostin gene promoter driving the expression of shRNA targeting the THBS1-AS1 knockdown (AAV2/9–periostin promoter–shTHBS1-AS1) was designed and purchased from Jikai Co. The sequences of the shRNAs were for shTHBS1-AS1 GATTGCTCAGAACCCAGGA and ACATAAAATGAATGCAATTGTTGTTGTTAACTTGTTTATTG. Seven days after TAC/Sham, the AAV2/9–periostin promoter–shTHBS1-AS1 or scramble (1.5 ***×*** 10^11^ vg) was administered by tail vein injection. Three weeks after the injection, echocardiography and heart dissection were performed.

### qPCR for mRNA and lncRNA.

Total RNA was extracted from the mouse heart tissues or cells using TRIzol (Invitrogen). The relative expression levels of mRNAs were measured using a RT kit (Toyobo) with a SYBR Green Supermix kit (Bio-Rad). After a 40-cycle reaction on Bio-Rad CFX96 Real-Time system, the gene expressions were calculated and normalized to the GAPDH. The relative fold changes were calculated using the 2^–ΔΔCT^ method. The sequences of the primers used are displayed in Supplemental Table 4.

### Western blot analysis.

A Western blot of mice hearts and CFs was performed as previously described (60). The antibodies used in this study are as follows: Smad2/3 (CST, 8685S, 1:1,000), phospho-SMAD2 (CST, 18338, 1:1,000), phospho-SMAD3 (CST, 9520, 1:1,000), and β-actin (Abcam, ab119716, 1:1,000). ImageJ software was used to quantify the intensities of the protein bands.

### Statistics.

The Shapiro-Wilk normality test was performed to detect deviations from normality. Data are presented as mean ± SEM. The data that conformed to normal distribution were analyzed using an unpaired 2-tailed Student’s *t* test or 1-way ANOVA with Bonferroni post hoc tests. The nonnormally distributed data were analyzed using the Wilcox or Kruskal-Wallis rank sum test. GraphPad Prism 7 and SPSS 22.0 software were used for all statistical analyses. *P* < 0.05 was considered significant.

### Study approval.

Animal experiments were approved by the animal ethics committee of West China Hospital, Sichuan University (no. 2018162A).

## Author contributions

JZ designed the research studies, performed the experiments, collected the data, and drafted the manuscript, and GT performed the experiments, collected the data, and revised the manuscript; order of first authors was decided based on these contributions. YQ, JZ, QK, and FH designed the statistical analysis strategy and performed the statistical analyses. JZ, GT, QK, FH, JL, WW, YT, ZZ, and XL contributed to the discussion. JZ, YT, ZZ, and XL edited the manuscript. YT, ZZ, and XL supervised the whole project. All of the authors reviewed and approved the final version of the manuscript.

## Supplementary Material

Supplemental data

## Figures and Tables

**Table 1:**
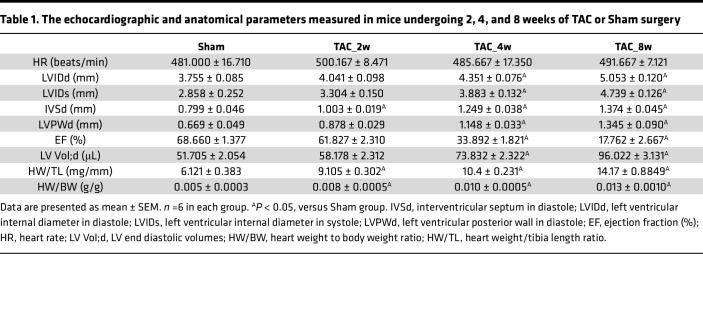
The echocardiographic and anatomical parameters measured in mice undergoing 2, 4, and 8 weeks of TAC or Sham surgery

## Version 1 02/14/2023

In-Press Preview

## Version 2 03/22/2023

Electronic publication
