# Supplementary material for: The long noncoding RNA THBS1-AS1 promotes cardiac fibroblast activation in cardiac fibrosis by regulating TGFBR1
Source: JCI Insight. 2023 Mar 22;8(6):e160745. doi: 10.1172/jci.insight.160745 (PMC10070117; doi:10.1172/jci.insight.160745)
Supplement: Supplemental data [file jciinsight-8-160745-s164.pdf]

Supplemental Tables:

Supplemental Table 1. Target sequences of siRNA

Names	Sequences
si-Nc	GGCUCUAGAAAAGCCUAUGC
Mouse-si-THBS1-AS1_1	TTCTAGACGAGGCTTCTTA
Mouse-si-THBS1-AS1_2	AGCAGGAAAAGTTGAAACA
Mouse-si-THBS1-AS1_3	TTGAATTCCTGCAGTCTTA
Mouse-ASO-THBS1-AS1	TCCTTCTAGACGAGGCTTCT
Mouse-si-MEOX1	GAGACGGAGAAGAAATCAT
Mouse-si TGFBR1_1	GCCATAACCGCACTGTCAT
Mouse-si TGFBR1_2	GCTATTGCCCATAGAGATT
Mouse-si TGFBR1_3	GCTGTTCTATTGGTGGAAT
Human-si-THBS1-AS1_1	CCACAGGCAACAATTAAGA
Human-si-THBS1-AS1_2	GTTCAAGAAACCTCTGCTT
Human-si-THBS1-AS1_3	GACTGCAGCATTCAGTAAA

Supplemental Table 2. Sequence of RNA FISH probe targeting THBS1-AS1

FISH-Probe	Sequence (5′-3′)
THBS1-AS1	CCCTGGCGACACCTGCGAATCCCTCGCCACATCGGCTGG
	AAAGATATCGGCTGGAAAGATTTCACTGCATACAHATCG
Nc	TGCTTTGCACGGTAACGCCTGTTTT

Supplemental Table 3. Primers for vector construction

Name	Primer sequence 5’ →3’
Psi-CHECK-THBS1-AS1-W-F	CGCTCGAGTGACTACAGTGAAATTGAATTCCTG
Psi-CHECK-THBS1-AS1-W-R	CAGCGGCCGCCTAGAAGGAGGCTGTGTTTCA
Psi-CHECK-THBS1-AS1-MUT-F	CGCTCGAGTGACTACAGTGAAATTGAATTCCTG
Psi-CHECK-THBS1-AS1-MUT-R	CAGCGGCCGCCTAGAAGGAGGCTGTGTTTCA
Psi-CHECK-TGFBR1-W-F	CGCTCGAGTTGAGGCCCTGTGTGGG
Psi-CHECK-TGFBR1-W-R	CAGCGGCCGCGGGCAGATTTTGGTGTGCA
Psi-CHECK-TGFBR1-MUT-F	CGCTCGAGTTGAGGCCCTGTGTGGG
Psi-CHECK-TGFBR1-MUT-R	CAGCGGCCGCGGGCAGATTTTGGTGTGCA

Notes: The nucleotides with underline are the restriction enzyme cutting sites.

Supplemental Table 4. Sequences of q-PCR primers

Genes	Primer sequence (5′-3′)
Mouse	
POSTN	F: 5′TGGTATCAAGGTGCTATCTGCG3′
	R: 5′AATGCCCAGCGTGCCATAA3′
CTGF	F: 5′GGACACCTAAAATCGCCAAGC3′
	R: 5′ACTTAGCCCTGTATGTCTTCACA3′
α-SMA	F: 5′GGACGTACAACTGGTATTGTGC3′
	R: 5′TCGGCAGTAGTCACGAAGGA3′
GAPDH	F: 5′CCTCGTCCCGTAGACAAAATG3′
	R: 5′TGAGGTCAATGAAGGGGTCGT3′
Gm13054	F: 5′ACTTCAGCACCAAGGACAGAC3′
	R: 5′TGAGGACTATGACGTGAGGC3′
Gm9913	F: 5′GAACAAGGCGAGTTATCTGAAC3′
	R: 5′AGGACACGATGCGCTGAAAG3′
Gm29233	F: 5′AATCTTTCCAGCCGATGTGG3′
	R: 5′CCAGGTAAGAAGCCTCGTCTA3′
D030025P21Rik	F: 5′GTAAGGGCTGTATGGCGTGA3′
	R: 5′GTGCCAAAGAGGGTGTTGCT3′
Dnm3os	F: 5′CCCATGACCACCCAACAGAA3′
	R: 5′CCCTGAACGGGGTACATTCC3′
TGFBR1	F: 5′TCTGCATTGCACTTATGCTGA3′
	R: 5′AAAGGGCGATCTAGTGATGGA3′
Human	
POSTN	F: 5′CCATGTTTATGGCACTCTGG3′
	R: 5′ACGTTGCTCTCCAAACCTCT3′
CTGF	F: 5′TCCGTACTCCCAAAATCTCC3′
	R: 5′AGTTGTAATGGCAGGCACAG3′
α-SMA	F: 5′AGGCGGTGCTGTCTCTCTAT 3′
	R: 5′AAGGAATAGCCACGCTCAGT3′
THBS1-AS1	F: 5′AGCTCCTGGGTCGTTTCATC3′
	R: 5′CTGTGGAGGAGGGGTACAGA3′
GAPDH	F: 5′GGAGCGAGATCCCTCCAAAAT3′
	R: 5′GGCTGTTGTCATACTTCTCATGG′

Supplemental Table 5. Mouse liver and kidney function.

	Sham	TAC_2w	TAC_4w	TAC_8w
ALT/(U/L)	121.11 ± 9.006	143.14 ± 15.153	210.41 ± 8.324*	276.63 ± 22.229*
AST/(U/L)	271.79 ± 32.839	299.16 ± 9.712	355.64 ± 11.398	668.13 ± 67.586*
TBil/(μmol/L)	29.67 ± 2.934	29.74 ± 3.527	66.17 ± 7.230*	74.17 ± 3.335*
Scr/(μmol/L)	58.60 ± 4.242	48.93 ± 8.675	151.88 ± 5.332*	203.57 ± 16.991*

Data are presented as mean ± SEM. n =6 in each group. *P<0.05, versus Sham group. ALT, aminotransferase; AST, aspartate aminotransferase; TBil, total bilirubin; Scr, serum creatinine

Supplemental Table 6. Top ten GO, KEGG and Reactome enrichment categories for differential gene expression between TAC_2w and sham group.

Category	ID	Term	FDR	Number
CC	GO:0031012	extracellular matrix	7.00E-37	185
CC	GO:0005578	proteinaceous extracellular matrix	4.59E-36	164
CC	GO:0044420	extracellular matrix component	1.83E-22	79
CC	GO:0005743	mitochondrial inner membrane	4.95E-17	146
CC	GO:0005604	basement membrane	7.15E-17	61
CC	GO:0019866	organelle inner membrane	5.94E-16	153
BP	GO:0030198	extracellular matrix organization	1.41E-15	92
BP	GO:0043062	extracellular structure organization	1.41E-15	92
CC	GO:0005581	collagen trimer	5.55E-15	48
MF	GO:1901681	sulfur compound binding	5.18E-14	93
KEGG	mmu04512	ECM-receptor interaction	1.64E-12	53
KEGG	mmu04510	Focal adhesion	3.96E-12	93
KEGG	mmu00280	Valine, leucine and isoleucine degradation	3.41E-09	33
KEGG	mmu00020	Citrate cycle (TCA cycle)	1.16E-08	24
KEGG	mmu05414	Dilated cardiomyopathy	5.53E-08	46
KEGG	mmu05410	Hypertrophic cardiomyopathy (HCM)	1.89E-06	41
KEGG	mmu00071	Fatty acid degradation	2.36E-06	26
KEGG	mmu05144	Malaria	2.95E-06	27
KEGG	mmu05146	Amoebiasis	1.58E-05	46
KEGG	mmu05412	Arrhythmogenic right ventricular cardiomyopathy (ARVC)	3.64E-05	35
Reactome	5991414	Extracellular matrix organization	1.54E-26	135
Reactome	5992182	Collagen formation	5.31E-17	51
Reactome	5992176	Degradation of the extracellular matrix	1.44E-15	65
Reactome	5992212	Collagen biosynthesis and modifying enzymes	1.18E-14	43
Reactome	5992282	ECM proteoglycans	5.91E-13	44
Reactome	5992181	Assembly of collagen fibrils and other multimeric structures	7.86E-13	33
Reactome	5991046	The citric acid (TCA) cycle and respiratory electron transport	1.04E-12	76
Reactome	5991045	Pyruvate metabolism and Citric Acid (TCA) cycle	2.34E-12	33
Reactome	5991413	Integrin cell surface interactions	3.00E-11	49
Reactome	5991299	Axon guidance	1.74E-09	113

GO, Gene Ontology; KEGG, Kyoto Encyclopedia of Genes and Genomes; MF, molecular function; BP, biological process; CC, cellular component.

Supplemental Table 7. Top ten GO, KEGG and Reactome enrichment categories for differential gene expression between TAC_4w and sham group.

Category	ID	Term	FDR	Number
CC	GO:0031012	extracellular matrix	5.54E-36	121
CC	GO:0005578	proteinaceous extracellular matrix	3.83E-33	106
CC	GO:0005581	collagen trimer	1.84E-20	40
CC	GO:0044420	extracellular matrix component	1.84E-16	48
BP	GO:0032787	monocarboxylic acid metabolic process	2.14E-13	101
MF	GO:1901681	sulfur compound binding	3.57E-12	57
BP	GO:0030335	positive regulation of cell migration	5.84E-12	82
BP	GO:0030198	extracellular matrix organization	9.79E-12	54
BP	GO:0043062	extracellular structure organization	9.79E-12	54
BP	GO:0040017	positive regulation of locomotion	9.79E-12	85
KEGG	mmu04512	ECM-receptor interaction	1.40E-12	38
KEGG	mmu00280	Valine, leucine and isoleucine degradation	9.59E-11	26
KEGG	mmu04510	Focal adhesion	6.05E-09	55
KEGG	mmu00640	Propanoate metabolism	2.33E-07	17
KEGG	mmu04974	Protein digestion and absorption	1.37E-06	26
KEGG	mmu05414	Dilated cardiomyopathy	1.46E-06	29
KEGG	mmu00650	Butanoate metabolism	2.23E-06	14
KEGG	mmu05410	Hypertrophic cardiomyopathy (HCM)	3.21E-06	27
KEGG	mmu00071	Fatty acid degradation	5.05E-06	18
KEGG	mmu00020	Citrate cycle (TCA cycle)	6.79E-06	15
Reactome	5991414	Extracellular matrix organization	1.02E-21	87
Reactome	5992182	Collagen formation	6.12E-16	38
Reactome	5992176	Degradation of the extracellular matrix	1.80E-15	47
Reactome	5992181	Assembly of collagen fibrils and other multimeric structures	2.05E-15	28
Reactome	5992212	Collagen biosynthesis and modifying enzymes	1.07E-14	33
Reactome	5991413	Integrin cell surface interactions	9.34E-14	38
Reactome	5992175	Collagen degradation	3.05E-11	29
Reactome	5992282	ECM proteoglycans	4.37E-10	29
Reactome	5991045	Pyruvate metabolism and Citric Acid (TCA) cycle	7.94E-10	23
Reactome	5991063	Mitochondrial Fatty Acid Beta-Oxidation	5.24E-09	13

GO, Gene Ontology; KEGG, Kyoto Encyclopedia of Genes and Genomes; MF, molecular function; BP, biological process; CC, cellular component.

Supplemental Table 8. JASPAR-predicted binding sites of MEOX1 within the THBS1-AS1 gene regulatory region with location, strand and relative binding score (with 80% cutoff).

Name	Score	Relative score	Sequence ID	Start	End	Strand	Predicted sequence
MA0661.1.MEOX1	9.744114	0.926373352	ENSMUST00000190311.1	1601	1610	+	gctaattgtc
MA0661.1.MEOX1	8.903158	0.909046834	ENSMUST00000190311.1	1090	1099	+	agtaatcatc
MA0661.1.MEOX1	8.8074045	0.907073987	ENSMUST00000190311.1	1014	1023	-	cctaatcatc
MA0661.1.MEOX1	6.07984	0.850876984	ENSMUST00000190311.1	1601	1610	-	gacaattagc
MA0661.1.MEOX1	6.0322323	0.849896102	ENSMUST00000190311.1	428	437	-	gctgattgac
MA0661.1.MEOX1	6.0125566	0.849490715	ENSMUST00000190311.1	1952	1961	+	actcatgaga
MA0661.1.MEOX1	5.5447206	0.839851722	ENSMUST00000190311.1	122	131	-	cctaactaaa
MA0661.1.MEOX1	5.268482	0.83416028	ENSMUST00000190311.1	1000	1009	-	gccaatcata
MA0661.1.MEOX1	4.706177	0.822574906	ENSMUST00000190311.1	1952	1961	-	tctcatgagt
MA0661.1.MEOX1	4.3848505	0.815954494	ENSMUST00000190311.1	1090	1099	-	gatgattact
MA0661.1.MEOX1	4.3068595	0.814347617	ENSMUST00000190311.1	1302	1311	+	agtaaatatg
MA0661.1.MEOX1	4.167354	0.811473337	ENSMUST00000190311.1	1011	1020	+	gctgatgatt
MA0661.1.MEOX1	3.9811862	0.807637651	ENSMUST00000190311.1	746	755	-	cctatttact
MA0661.1.MEOX1	3.7170448	0.802195451	ENSMUST00000190311.1	746	755	+	agtaaatagg
MA0661.1.MEOX1	3.6895893	0.801629774	ENSMUST00000190311.1	1093	1102	-	cctgatgatt
MA0661.1.MEOX1	3.6590042	0.800999619	ENSMUST00000190311.1	1014	1023	+	gatgattagg

Supplemental Figures:

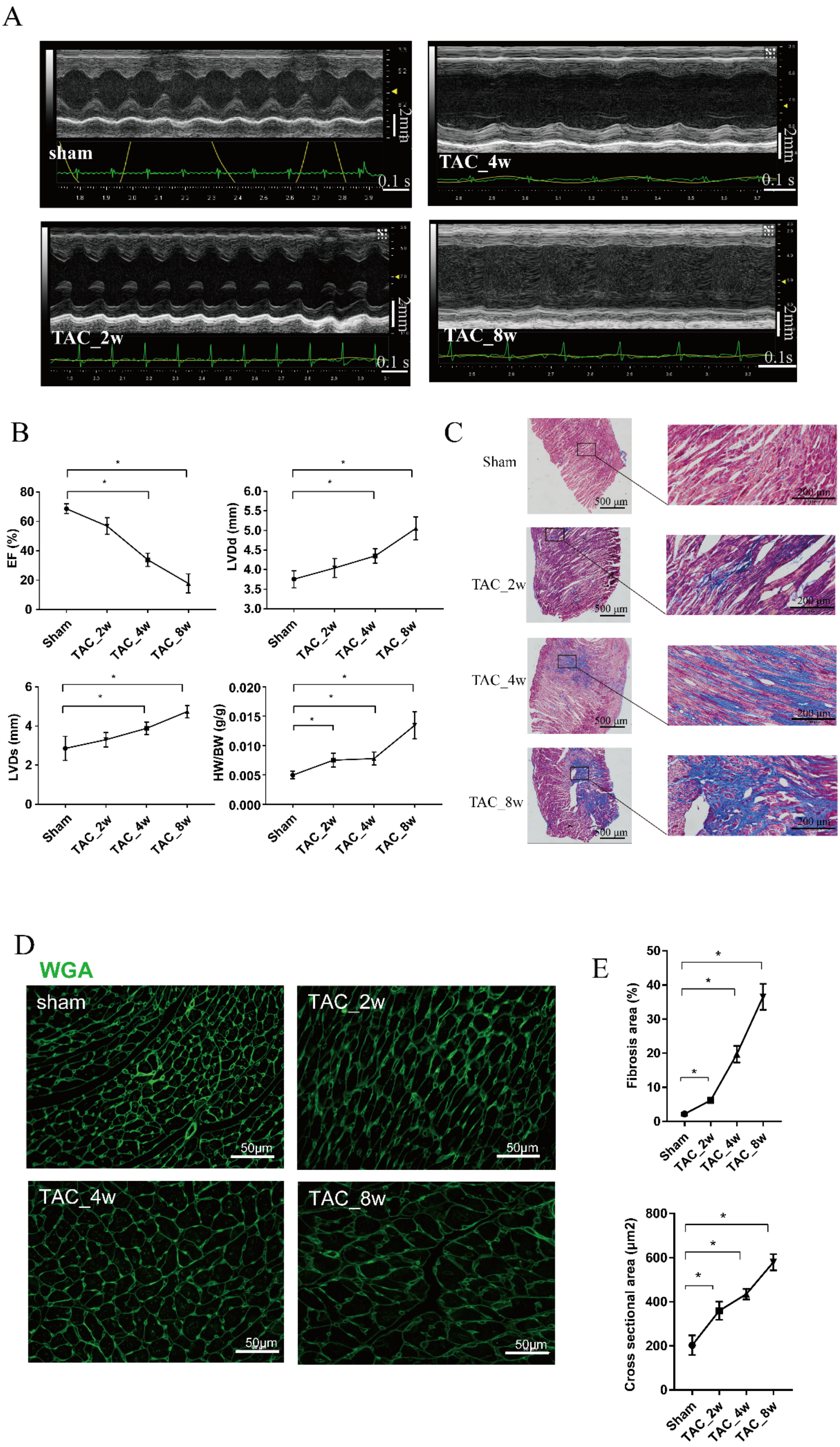

A
sham
TAC_4w
TAC_2w
TAC_8w
2mm
0.1 s
B
EF (%)
LVDd (mm)
LVDs (mm)
HW/BW (g/g)
Sham
TAC_2w
TAC_4w
TAC_8w
C
Sham
TAC_2w
TAC_4w
TAC_8w
500 μm
200 μm
D
WGA
sham
TAC_2w
TAC_4w
TAC_8w
50μm
E
Fibrosis area (%)
Cross sectional area (μm2)

Supplemental Figure 1. Evaluation of mouse cardiac fibrosis model. (A) Representative images of echocardiography from mice 2, 4 and 8 weeks after TAC and sham treatment. (B) Cardiac function indicators measured by echocardiography and anatomy index (LVEF (%), LVDd, LVDs and HW/BW) in 2, 4 and 8 weeks after TAC and sham treatment. (C) Representative images of Masson's Trichrome–stained sections of hearts following 2, 4 and 8 weeks after TAC. Blue denotes collagen fibers. (D) Representative WGA images of mouse hearts following 2, 4 and 8 weeks after TAC. (E) Quantification of cross section area (μm^2) estimated by WGA and fibrosis area (%) estimated by Masson trichrome staining. (n = 6 in each group). Unpaired, two-tailed t-test. ＊ indicates $P < 0.05$ versus sham.

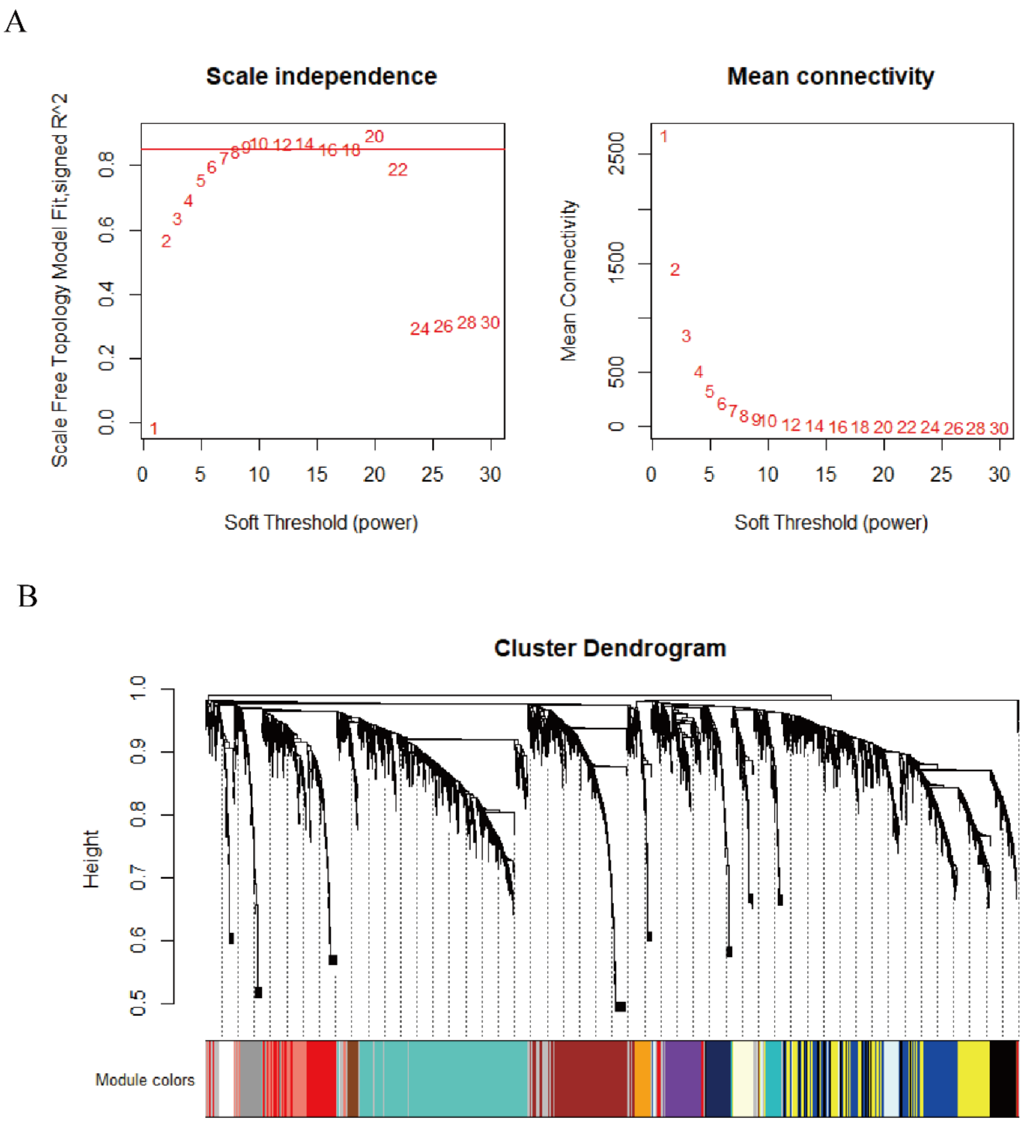

Supplemental Figure 2. Determination of soft-threshold power and modules in WGCNA (A) The calculation of the scale-free fit index and the mean connectivity with various soft-thresholding powers. (β). (B) The clustering dendrogram of genes. The gene clustering tree (dendrogram) obtained by average linkage hierarchical clustering.

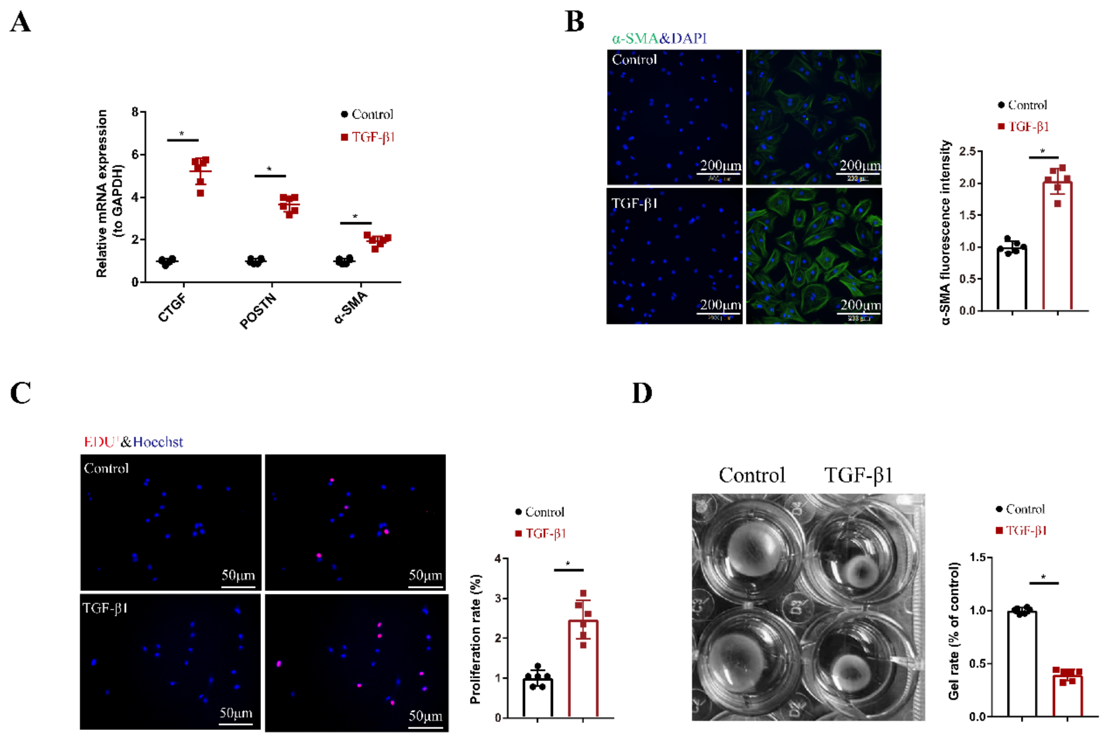

Supplemental Figure 3. TGF-β1–induced cardiac fibroblast activation. (A) mRNA expression of CTGF, α-SMA and POSTN in cardiac fibroblasts after TGF-β1-treatment. (B) Immunofluorescence images showing α-SMA expression in cardiac fibroblasts. (C) EdU staining used to detect cell proliferation. Cell nuclei are stained blue (DAPI), and EdU-positive nuclei are stained red. (D) Collagen gel contraction assessment after TGF-β1-treatment. Percentage contraction measured as gel size relative to culture area. n =6 in each group. Unpaired, two-tailed t-test. Results are presented as means ± standard error of the means; * indicates $p < 0.05$

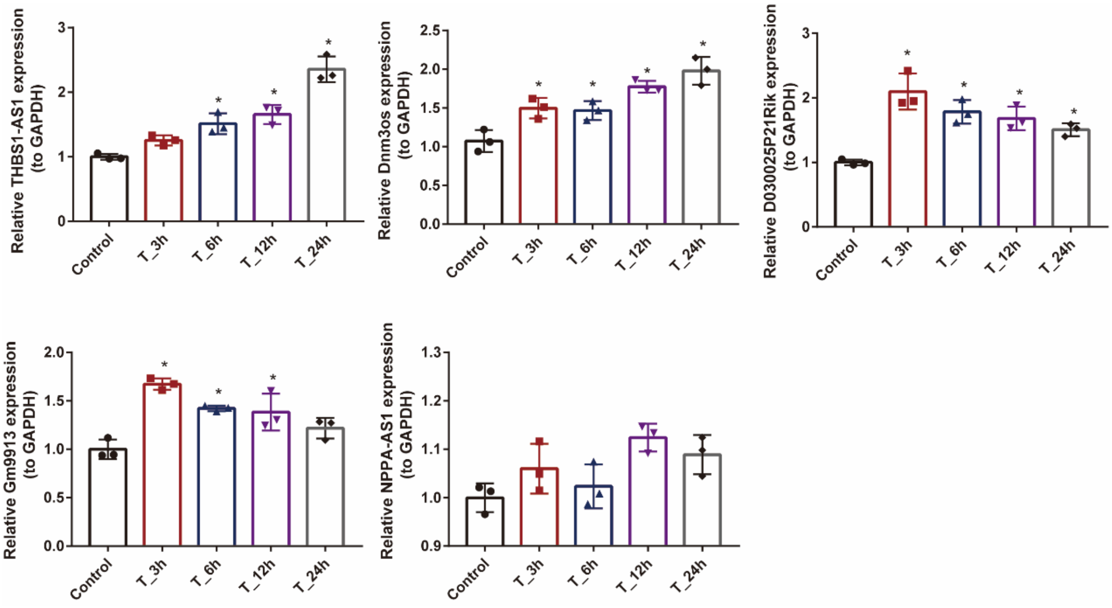

Supplemental Figure 4. Gene expression of lncRNA, THBS1-AS1, Dnm3os, D030025P21Rik, Gm9913 and NPPA-AS1, and stimulated with TGF-β1 for 3, 6, 12 and 24 h. (n=3). Unpaired, two-tailed t-test. Results are presented as means ± standard error of the means; * indicates $p < 0.05$ versus control

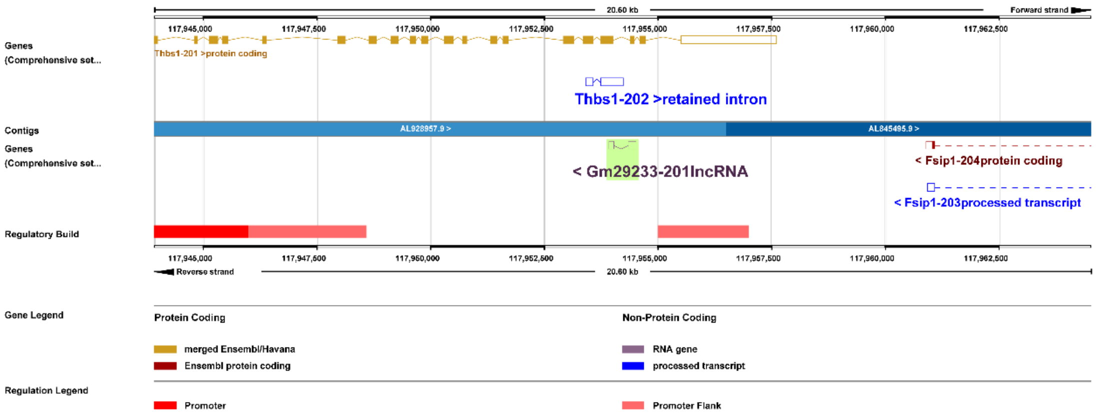

Supplemental Figure 5. Genome browser view of the region of lncRNA Gm29233 (THBS1-AS1).

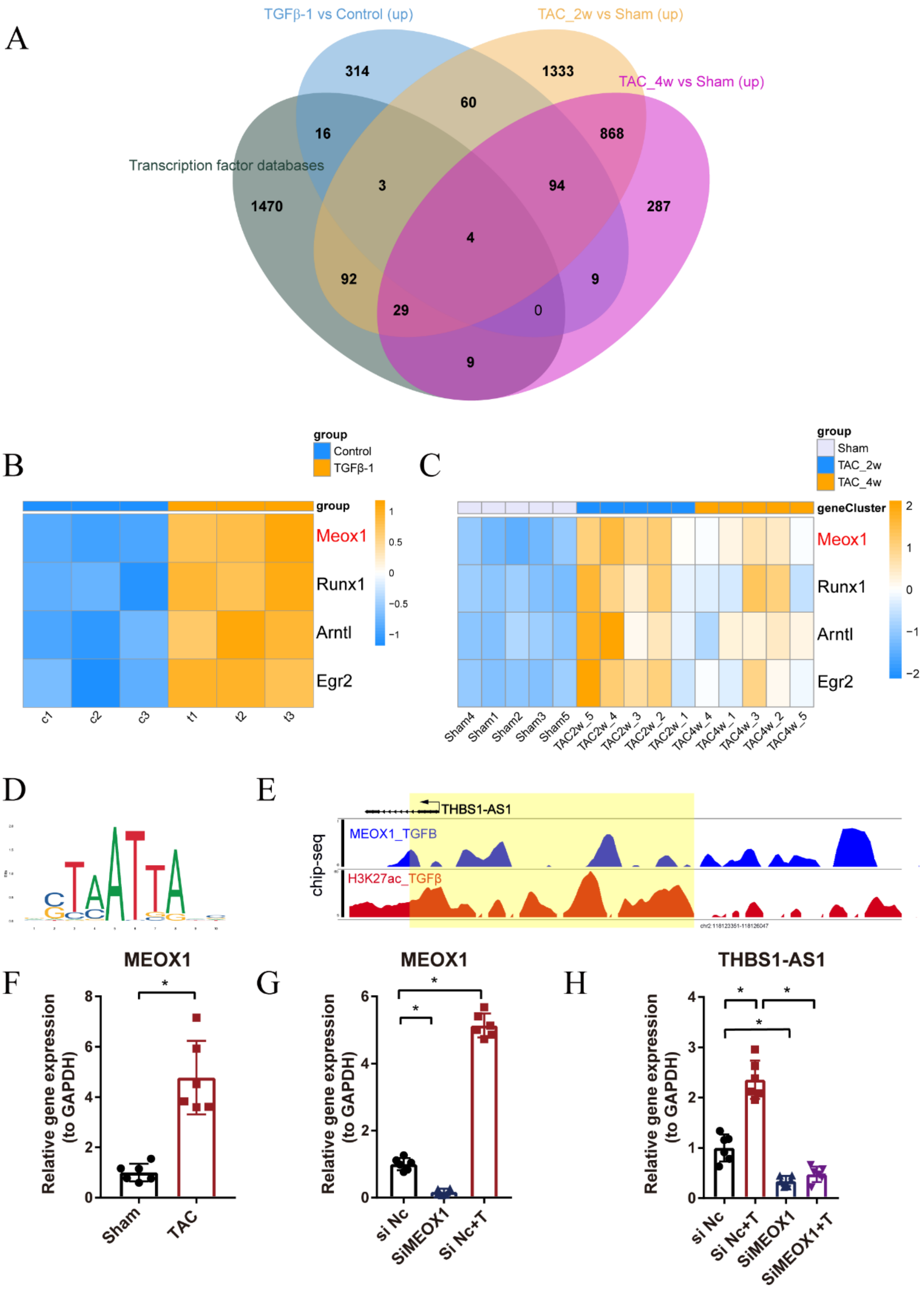
A
TGFβ-1 vs Control (up)
TAC_2w vs Sham (up)
TAC_4w vs Sham (up)
Transcription factor databases
314
1333
60
868
16
3
94
287
1470
4
92
9
29
0
9
B
group
Control
TGFβ-1
Meox1
Runx1
Arntl
Egr2
c1
c2
c3
t1
t2
t3
C
group
Sham
TAC_2w
TAC_4w
geneCluster
Meox1
Runx1
Arntl
Egr2
Sham4
Sham1
Sham2
Sham3
Sham5
TAC2w_5
TAC2w_4
TAC2w_3
TAC2w_2
TAC2w_1
TAC4w_4
TAC4w_1
TAC4w_3
TAC4w_2
TAC4w_5
D
E
THBS1-AS1
chip-seq
MEOX1_TGFB
H3K27ac_TGFβ
F
MEOX1
Relative gene expression (to GAPDH)
Sham
TAC
G
MEOX1
Relative gene expression (to GAPDH)
si Nc
SiMEOX1
Si Nc+T
H
THBS1-AS1
Relative gene expression (to GAPDH)
si Nc
Si Nc+T
SiMEOX1
SiMEOX1+T

Supplemental Figure 6. Evaluation of mouse cardiac fibrosis model. (A) Venn diagram illustrating the distribution of up-regulated genes in mouse transcription factor database (TFDB), TGFβ1 vs Control, TAC_2w vs Sham, TAC_4w vs Sham. (B) Expression pattern of four transcription factors, Meox1, Runx1, Arntl and Egr2 among the control and TGFβ1 group. (C) Expression pattern of Meox1, Runx1, Arntl and Egr2 among the sham, TAC_2w and TAC_4w group. (D) JASPAR prediction motif of MEOX1 DNA binding sequence between−2000 bp and +100 bp around the Transcription Starting Site (TSS) of the THBS1-AS1. (E) Coverage of indicated MEXO1/H3K27AC ChIP–seq with TGFβ treatment in cardiac fibroblasts at the THBS1-AS1 locus (GSE15582). (F) Gene expression of MEOX1 in mouse hearts by q-PCR. (n = 6). Unpaired, two-tailed t-test. (G) Gene expression of MEOX1 in cardiac fibroblasts by q-PCR (n = 6). (H) Gene expression of THBS1-AS1 in cardiac fibroblasts by q-PCR. One-way ANOVA followed by Bonferroni post hoc test.

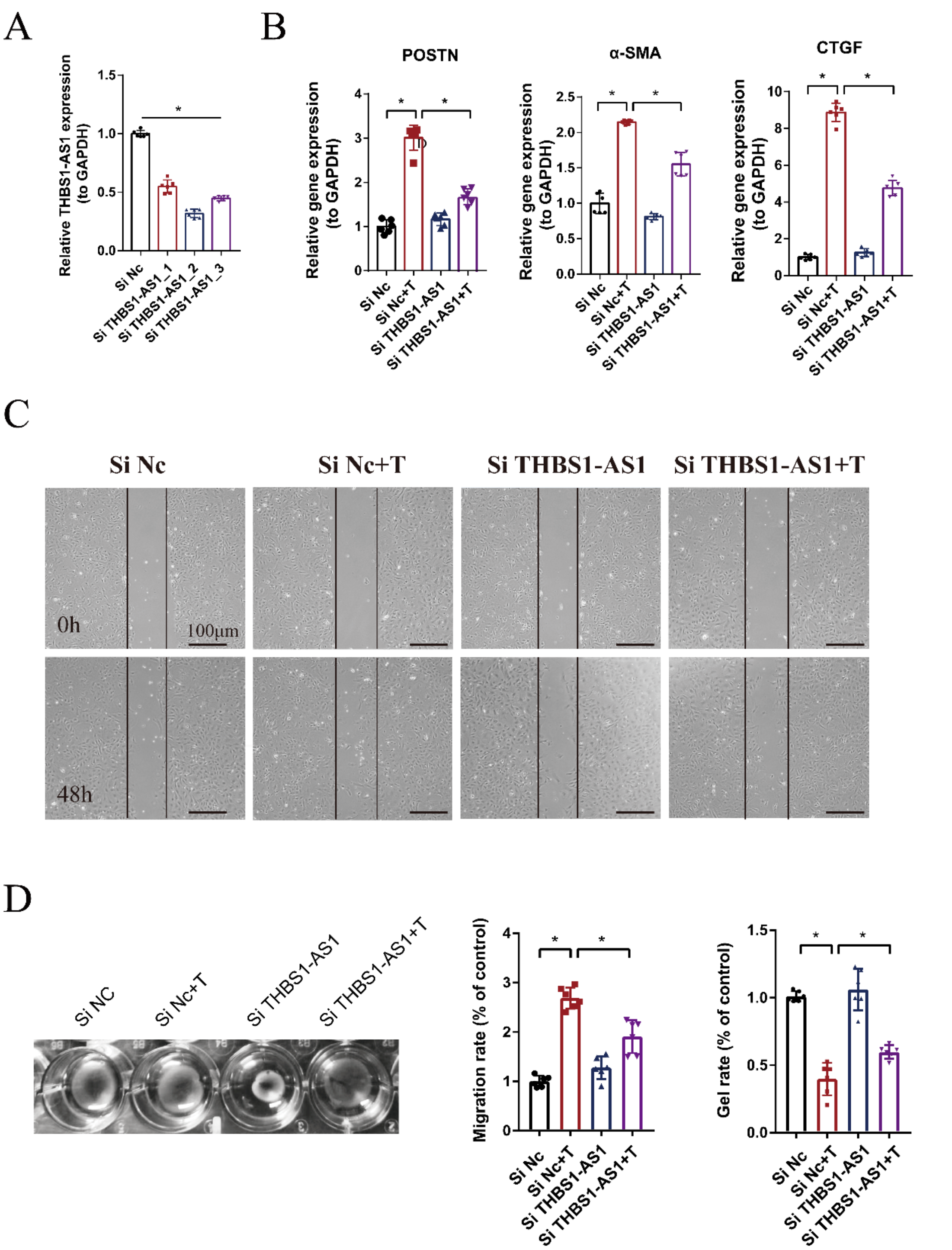

Supplemental Figure 7. Knockdown of THBS1-AS1 attenuated fibrogenesis in cardiac fibroblast activation. (A) The efficiency of Si-THBS1-AS1 silencing in cardiac fibroblasts. (n = 6). An unpaired, two-tailed t-test. (B) The gene expression of CTGF, α-SMA and POSTN in cardiac fibroblasts by q-PCR. (n = 6). A one-way ANOVA followed by a Bonferroni post hoc test. (C) *In vitro* scratch wound assays of cardiac fibroblasts after transfection with Si THBS1-AS1; (D) Collagen gel contraction assessment after transfection with si THBS1-AS1. n = 6 in each group; One-way ANOVA followed by Bonferroni post hoc test. * indicates $p < 0.05$.

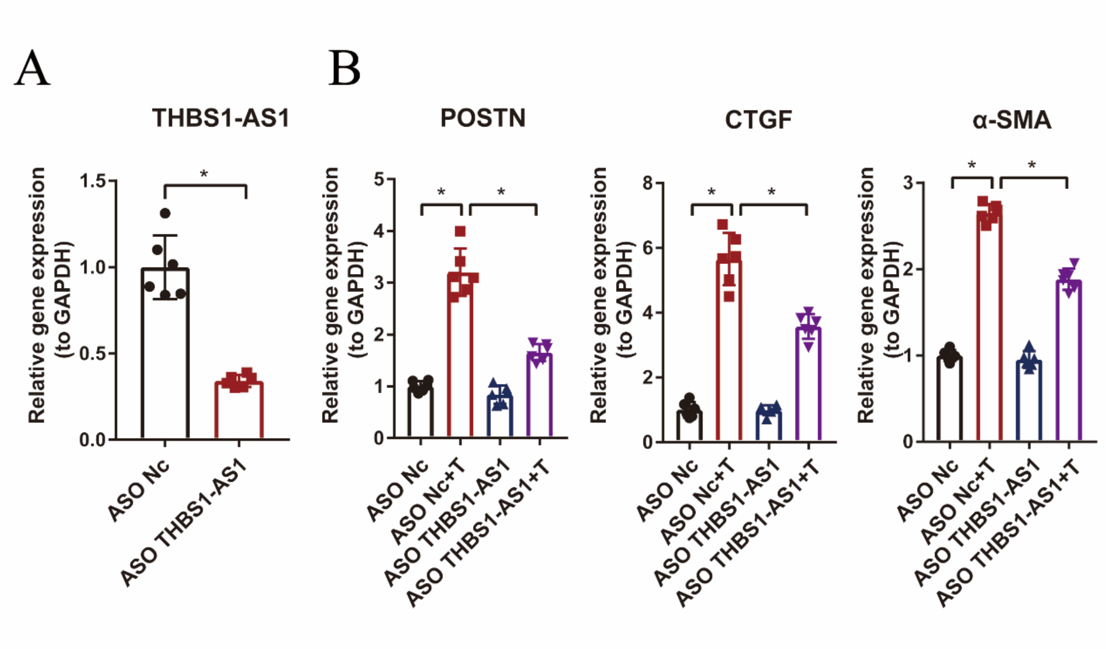

Supplemental Figure 8. Knockdown of THBS1-AS1 mediated by ASO attenuated fibrogenesis in cardiac fibroblast activation. (A) Efficiency of THBS1-AS1 silencing in cardiac fibroblasts. (n = 6). Unpaired, two-tailed t-test; (B) Gene expression of CTGF, α-SMA and POSTN in cardiac fibroblasts by q-PCR (n = 6). One-way ANOVA followed by Bonferroni post hoc test. * indicates p < 0.05.

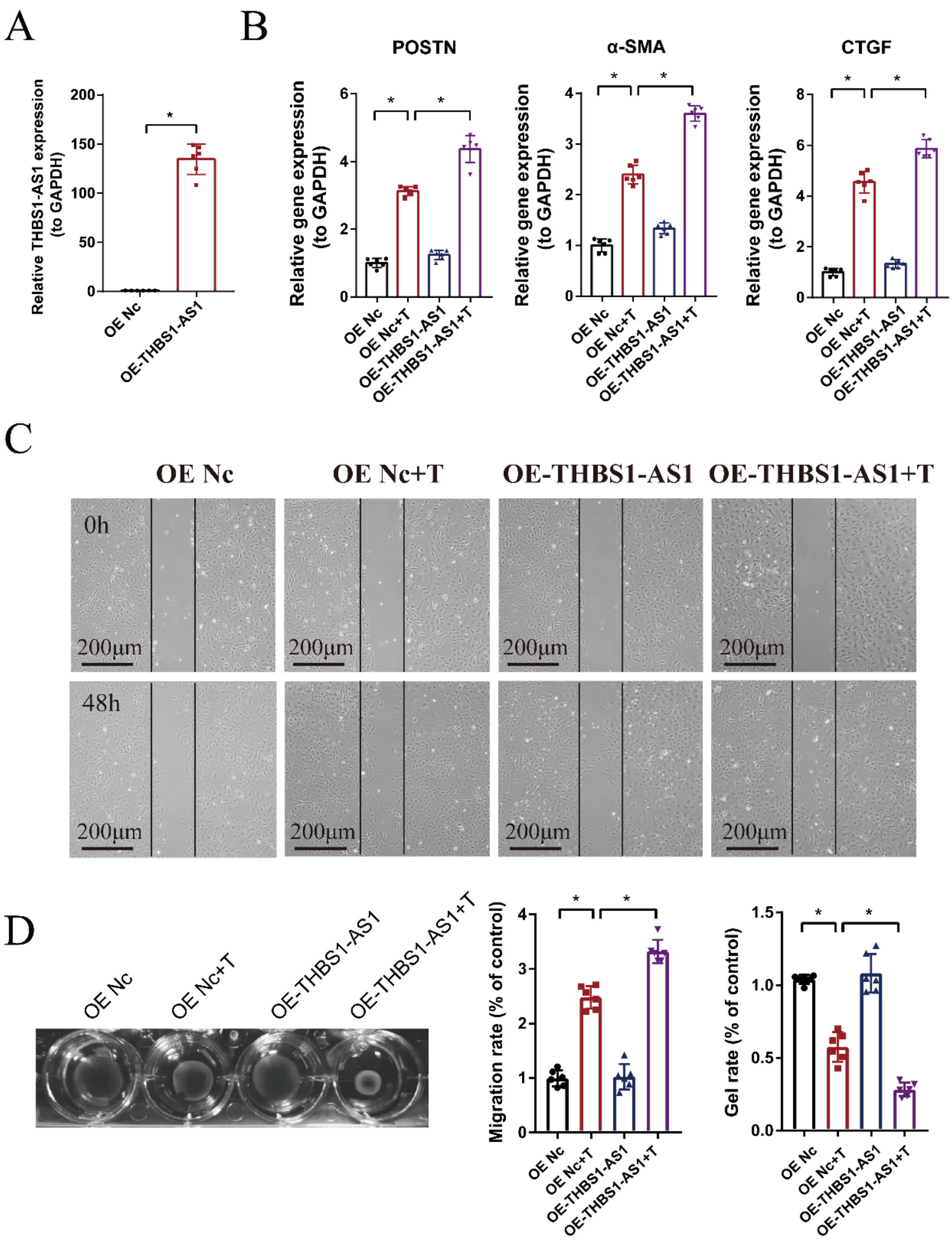

Supplemental Figure 9. Overexpression of THBS1-AS1 aggravated fibrogenesis in cardiac fibroblast activation. (A) The efficiency of the adenoviral overexpression of THBS1-AS1. (n = 6). An unpaired, two-tailed t-test. (B) The gene expression of CTGF, α-SMA, and POSTN in cardiac fibroblasts by q-PCR. (n = 6). A one-way ANOVA followed by a Bonferroni post hoc test. (C) In vitro scratch wound assays of cardiac fibroblasts infected by adenovirus to overexpression THBS1-AS1; (D) Collagen gel contraction assessment infected by adenovirus to overexpression THBS1-AS1. n = 6 in each group; One-way ANOVA followed by Bonferroni post hoc test. * indicates $p < 0.05$.

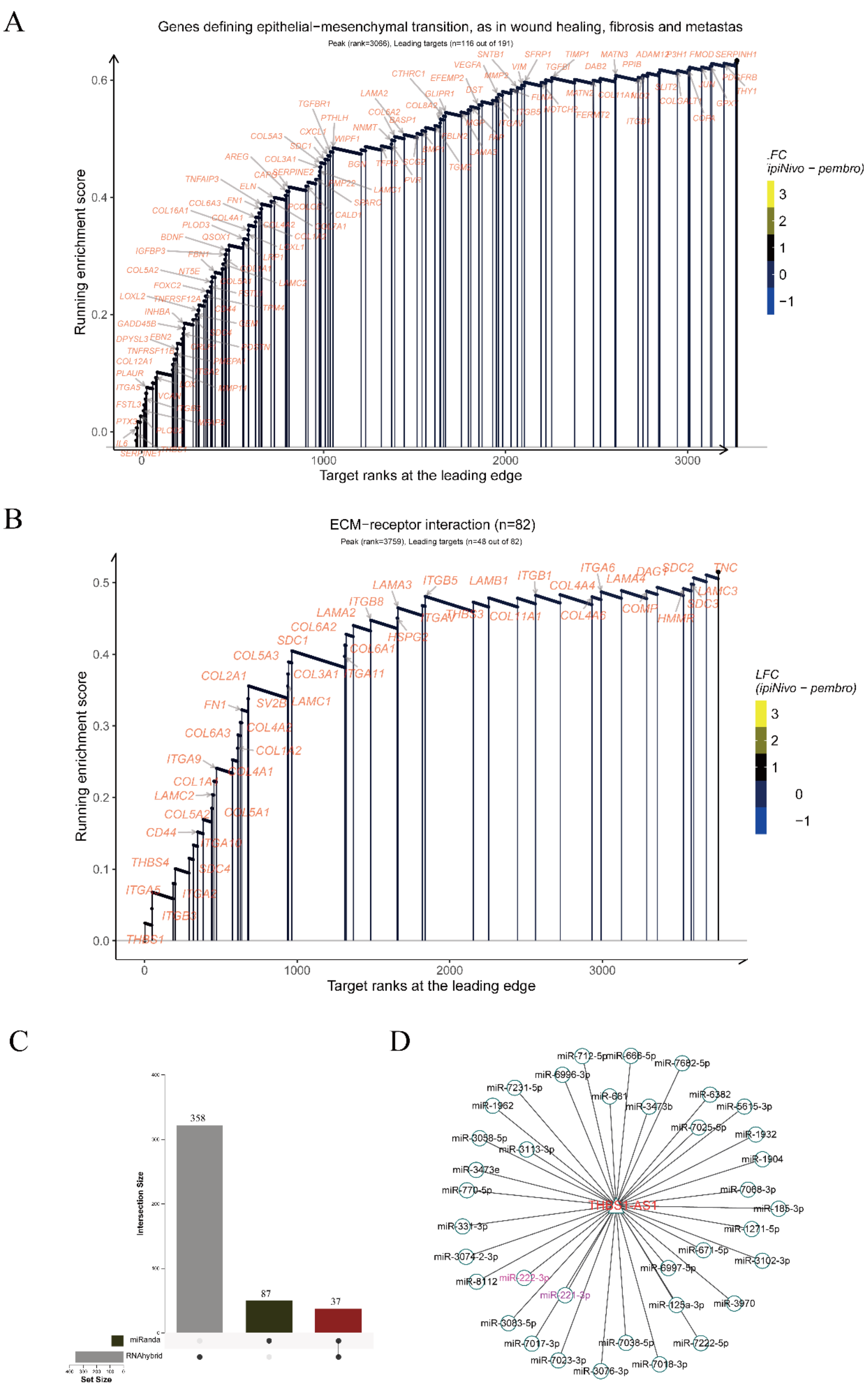
A
Genes defining epithelial−mesenchymal transition, as in wound healing, fibrosis and metastas
Peak (rank=3066), Leading targets (n=116 out of 191)
Running enrichment score
Target ranks at the leading edge
LFC
(ipiNivo − pembro)
B
ECM−receptor interaction (n=82)
Peak (rank=3759), Leading targets (n=48 out of 82)
Running enrichment score
Target ranks at the leading edge
LFC
(ipiNivo − pembro)
C
358
87
37
Intersection Size
miRanda
RNAhybrid
Set Size
D
THBS1-AS1
miR-222-3p
miR-221-3p

Supplemental Figure 10. The potential regulation mechanisms of THBS1-AS1 in the fibrosis process and potential THBS1-AS1-miRNA interactions predicted by bioinformatics method. (A) The enrichment plots showing Rank-based genes related to the epithelial-mesenchymal transition, wound healing, fibrosis, and the metastasis signaling pathway, and (B) the extracellular matrix receptor interaction pathway in pressure overload induced cardiac fibrosis. (C) Putative binding miRNA predicted by the RNAhybrid and miRanda algorithm. (D) The interactions between THBS1-AS1 and the predicted miRNA.

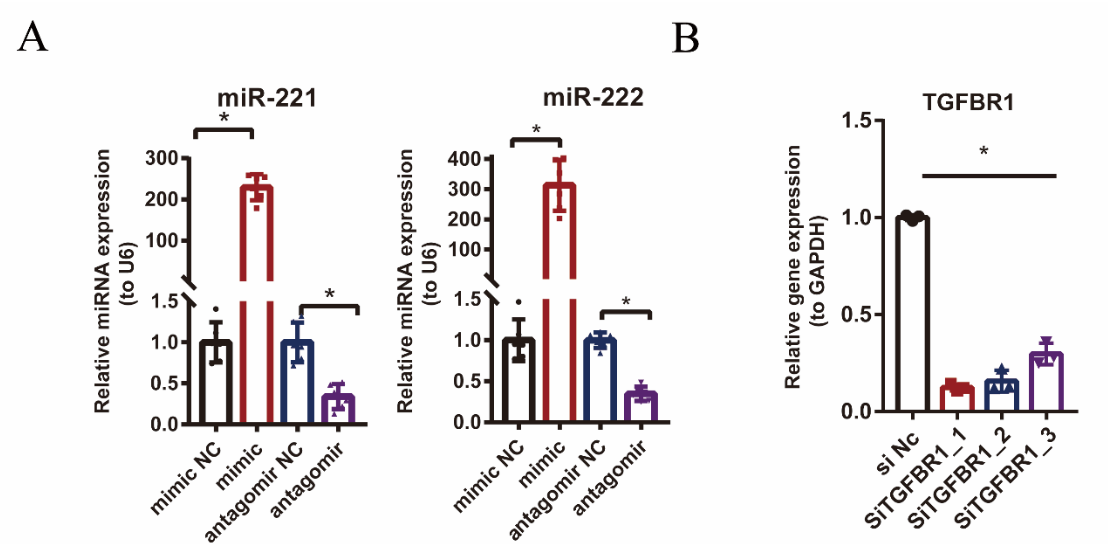

Supplemental Figure 11. (A) The expression of miR-221 and miR-222 in cardiac fibroblasts after antagomiR-221/222 or miR-221/222 mimic treatment. (n = 6). (B) Efficiency of si-TGFBR1 silencing in cardiac fibroblasts by q-PCR. (n = 3). Unpaired, two-tailed t-test. * indicates $p < 0.05$.

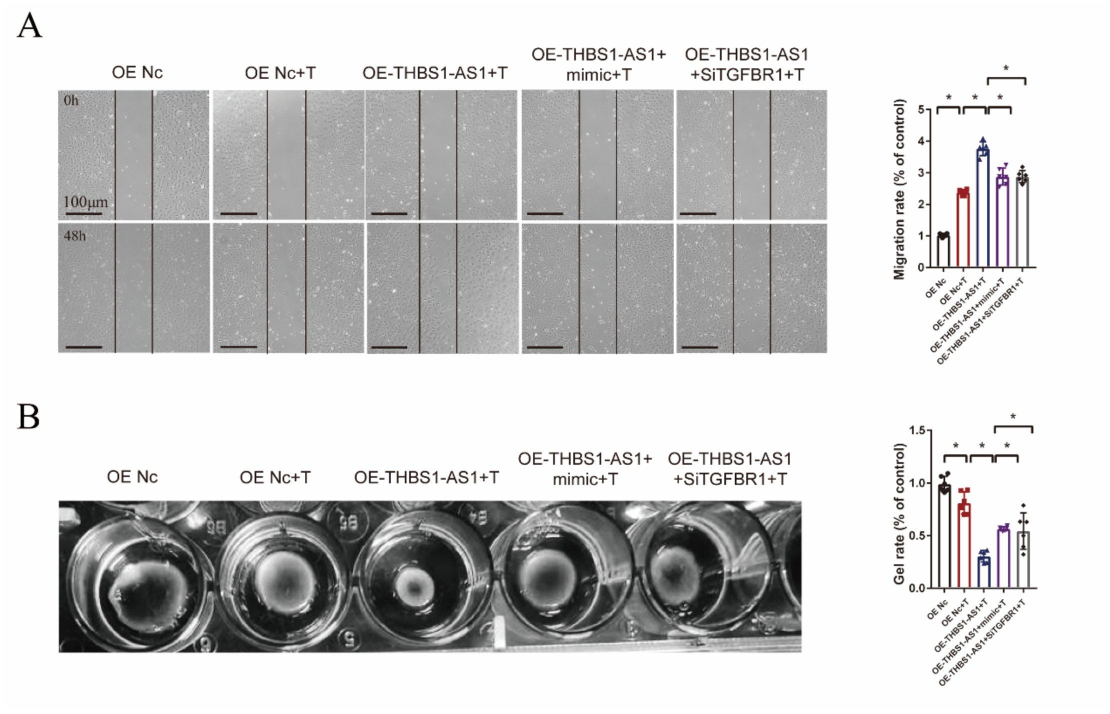

Supplemental Figure 12. THBS1-AS1 mediates the pro-fibrotic function via the miR-221/222-TGFBR1 axis. (A) *In vitro* scratch wound assays and (B) Collagen gel contraction assessment of cardiac fibroblasts transfected with adenovirus-mediated OE-THBS1-AS1 alone or miR-221/222 mimic and OE-THBS1-AS1 or siRNA-mediated TGFBR1 knockdown and OE-THBS1-AS1 treatment with/without TGF-β1 stimulation. (n = 6). One-way ANOVA followed by Bonferroni post hoc test. * indicates $p < 0.05$.

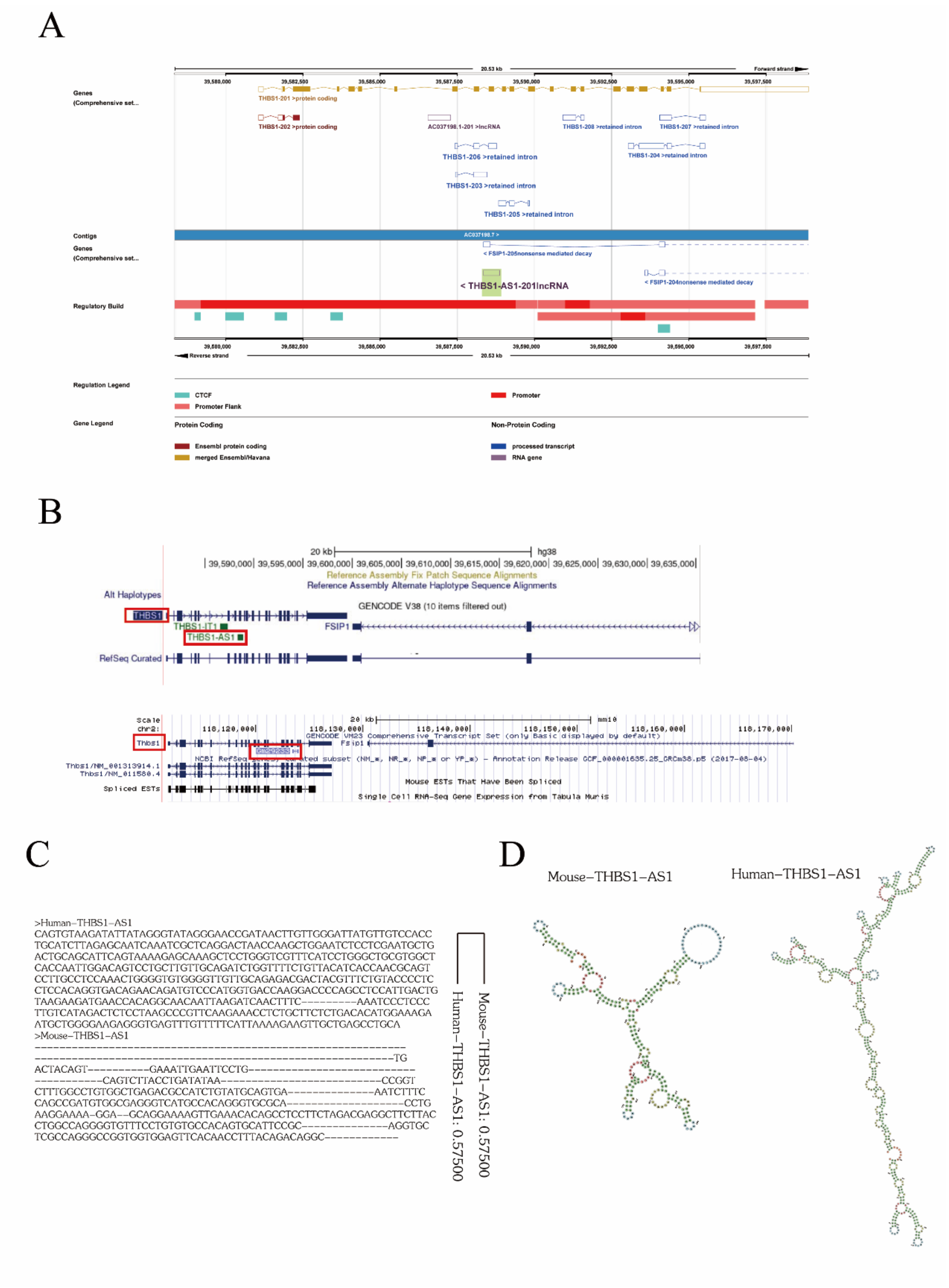

Supplemental Figure 13. (A) Genome browser view of the region of lncRNA human-THBS1-AS1. (B) The location of lncRNA mouse-THBS1-AS1 and human-THBS1-AS1 obtained from the UCSC Genome Browser (http://genome.ucsc.edu/cgi-bin/hgGateway). (C) The sequences of lncRNA mouse-THBS1-AS1 and human-THBS1-AS1 aligned using the MAFFT version 6.707b software. (D) The RNA structures of mouse-THBS1-AS1 and human-THBS1-AS1 were predicted by the RNA fold and Forna database.

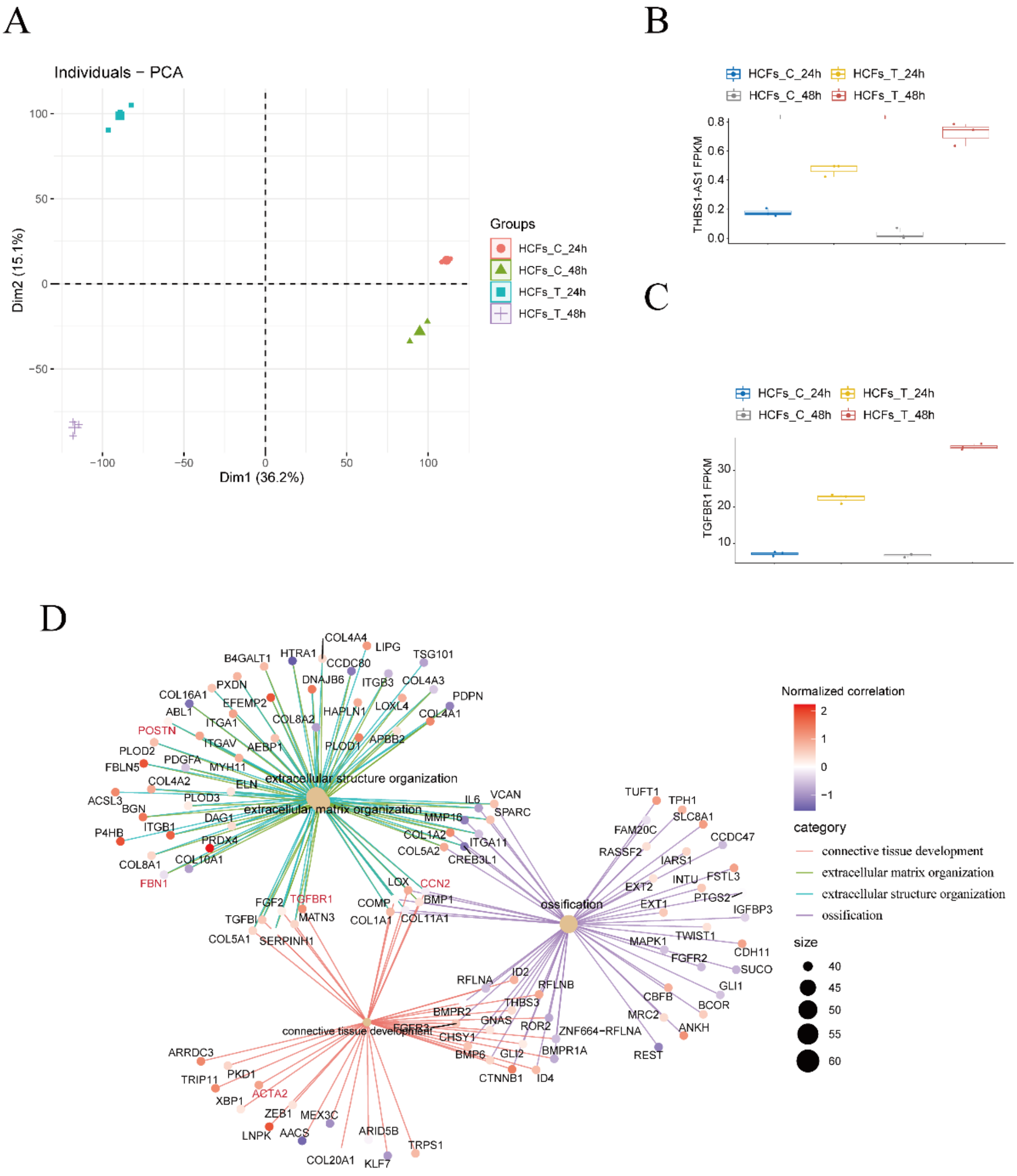

Supplemental Figure 14. (A) PCA analysis of the human cardiac fibroblasts after 24 and 48 hours of TGF-β1 stimulation (GSE152250). (B) The normalized FPKM expression of THBS1-AS1 and (C) TGFBR1 in the human cardiac fibroblasts after 24 and 48h of TGF-β1 stimulation. (D) The diagram of the enrichment pathway interaction network for genes significantly associated with THBS1-AS1.

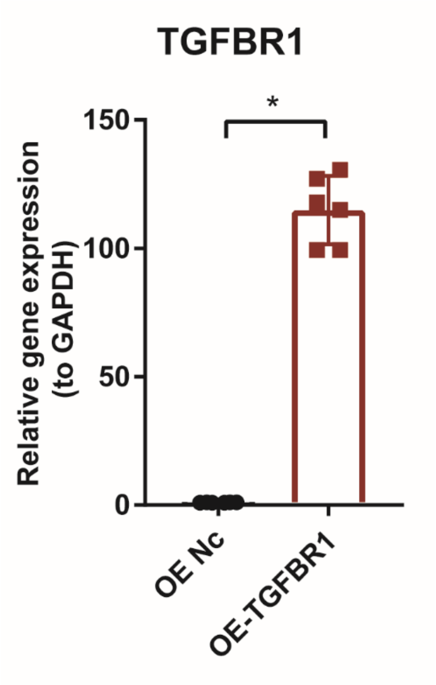

Supplemental Figure 15. The overexpression efficiency of TGFBR1 overexpression plasmid was assessed by q-PCR (n = 6). Unpaired, two-tailed t-test.

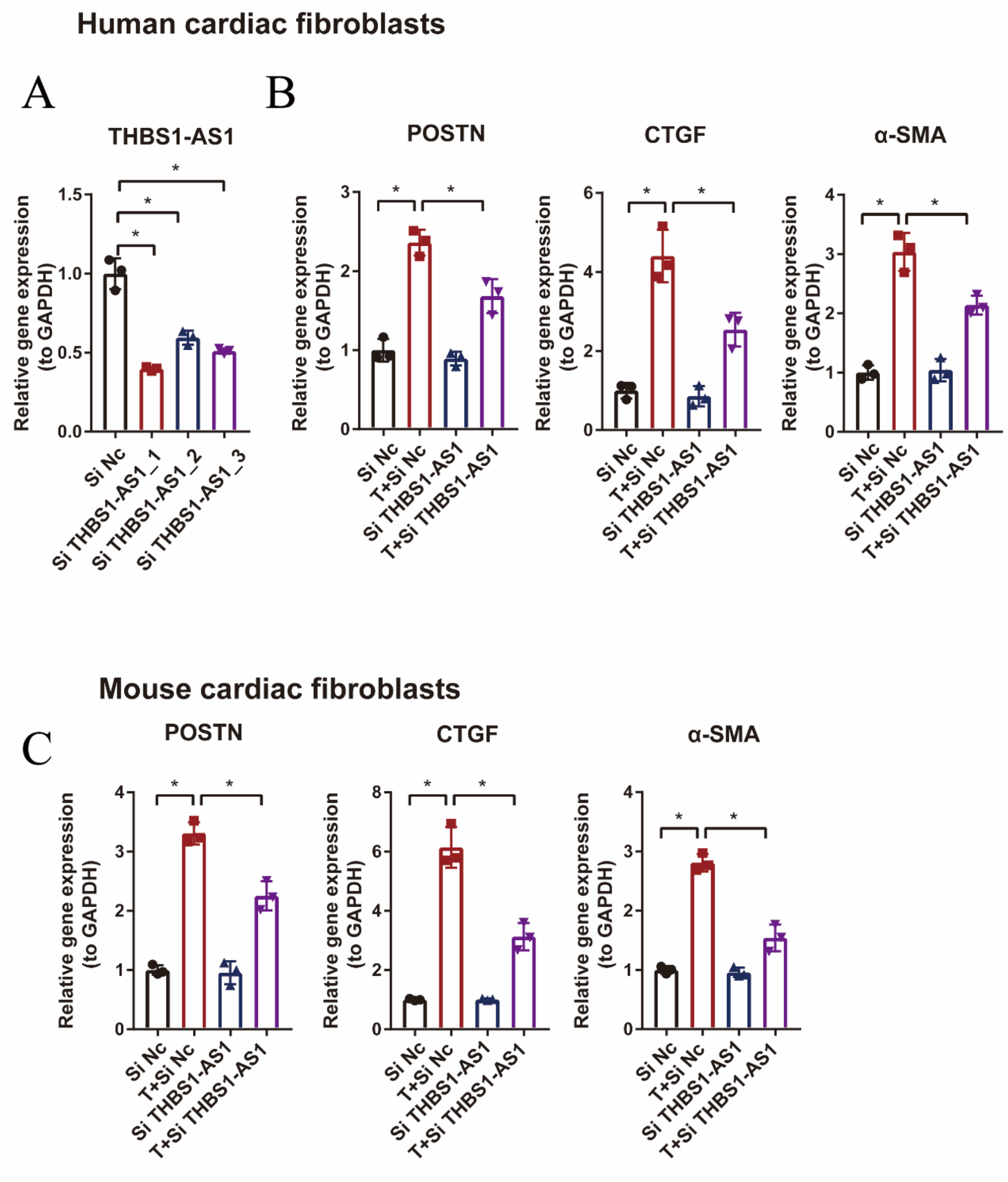

Supplemental Figure 16. Pre-conditioned with TGF-β1 for 24 hours prior to knockdown of THBS1-AS1 treatment attenuated fibrogenesis in human and mouse cardiac fibroblast activation. (A) Efficiency of THBS1-AS1 silencing mediated by siRNA in cardiac fibroblasts. (n = 3). Unpaired, two-tailed t-test. (B-C) Gene expression of CTGF, α-SMA and POSTN in cardiac fibroblasts by q-PCR (n = 3). One-way ANOVA followed by Bonferroni post hoc test.

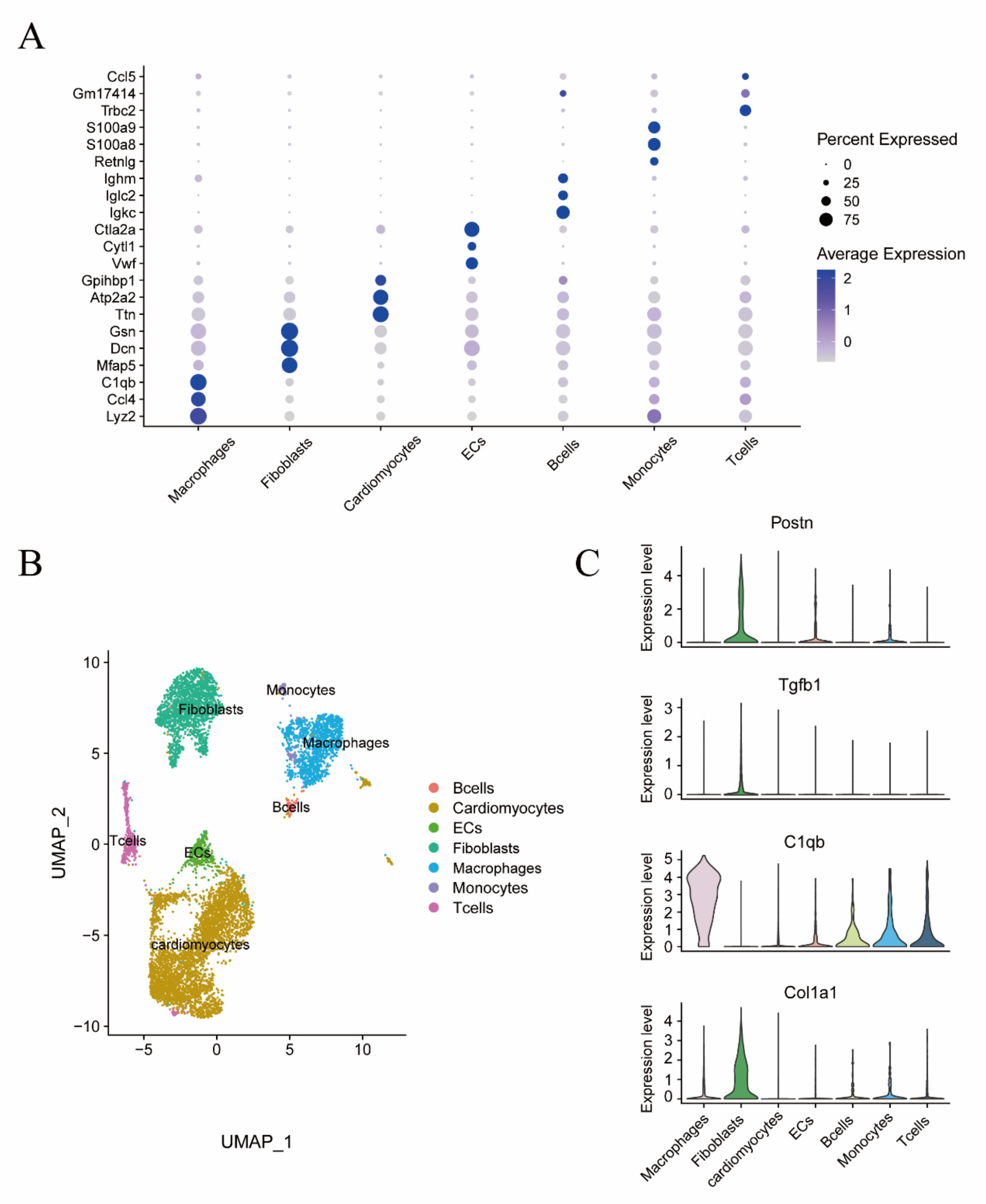

Supplemental Figure 17. Single-cell analysis of cardiac fibrosis in a mouse model of pressure overload (GSE120064). (A) Violin plot showing the top-3 markers per cluster in the scRNA-seq data. Total cells, n = 8161 in 7 clusters. (B) Uniform manifold approximation and projection (UMAP) plot of cells captured from hearts in colored by cluster. (C) Expression by cluster of POSTN, TGFB1, C1QB, COL1A1 shown in violin plots.

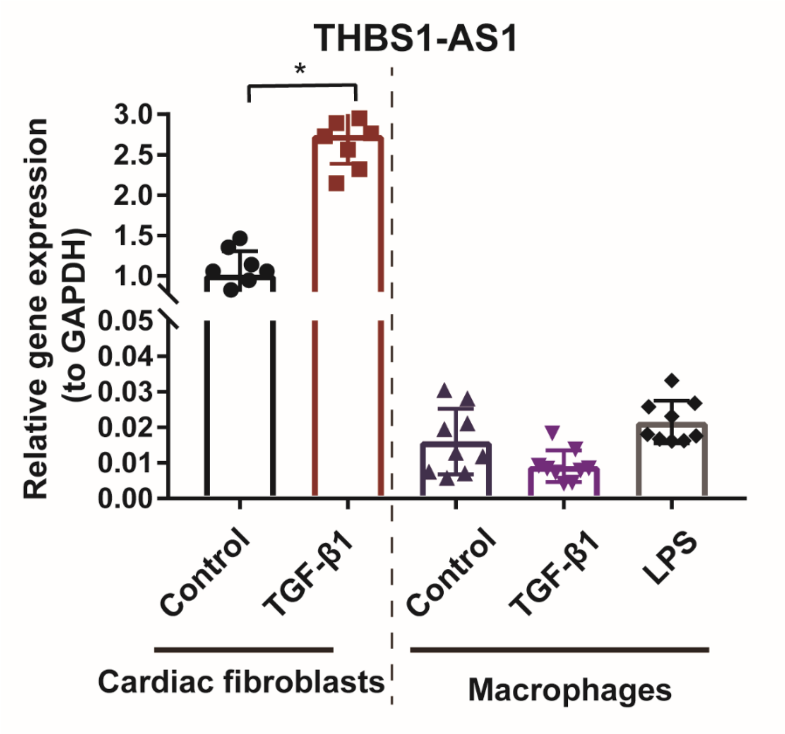

Supplemental Figure 18. Expression of THBS1-AS1 in mouse cardiac fibroblasts and RAW 264.7 macrophages treated with TGF-β1 (10 ng/ml) or LPS (100 ng/ml) by q-PCR (n = 9). Unpaired, two-tailed t-test in the left panel. One-way ANOVA followed by Bonferroni post hoc test in the right panel.
